# Root system architecture and drought adaptation: emerging tools and genetic insights

**DOI:** 10.3389/fpls.2026.1753086

**Published:** 2026-01-30

**Authors:** Vikender Kaur, Shashank Kumar Yadav, Bindu Yadav, Sukham Madaan, Munisha Kheralia, Viswanathan Chinnusamy

**Affiliations:** 1 Division of Germplasm Evaluation, Indian Council of Agricultural Research (ICAR)-National Bureau of Plant Genetic Resources, New Delhi, India; 2 Department of Botany and Plant Physiology, Chaudhary Charan Singh (CCS) Haryana Agricultural University, Hisar, Haryana, India; 3 Division of Plant Physiology, Indian Agricultural Research Institute (ICAR), New Delhi, India

**Keywords:** climate change, genetic regulation, lateral root density, non-invasive approaches, phytohormone crosstalk, QTLs, rhizotron, root depth

## Abstract

Strategic optimisation of Root System Architecture (RSA) represents a critical frontier for stabilising crop productivity amid increasingly unpredictable moisture-deficit regimes. Understanding key root traits underlying effective drought response is necessary to harness the genetic diversity associated with root growth patterns and environmental adaptations. Many functionally significant root architectural traits have been reported, and the mechanistic importance of some of the anatomical ideotypes, such as the increased metaxylem vessel diameter to reduce axial hydraulic resistance to maintain leaf water potential and change in root growth angle to promote geotropic deep-soil moisture foraging, are discussed in this review. Despite the identification of these characteristics, the knowledge gap in their integration into predictive breeding frameworks remains. This review addresses this fragmentation by critically evaluating how the bottleneck of the ‘phenotyping’ process is being broken down through non-invasive high-throughput phenotyping modalities. Dynamic root-soil interfaces can be spatio-temporally quantified *in situ* using non-destructive technologies such as X-ray computed tomography and MRI, which can detect developmental plasticity masked by destructive sampling. Artificial Intelligence (AI), especially Convolutional Neural Networks, enables automated extraction of high-dimensional topological parameters from complex digital rhizograms. Present review integrates recent advances in phenotyping with molecular regulatory mechanisms, bridging two traditionally disparate fields. By focusing on the DRO1/qSOR1 loci and ABA-auxin crosstalk, we establish critical connections between molecular regulation and field-scale architectural performance. The resulting multi-scale roadmap may help in targeted selection of climate-resilient cultivars to maximize resource use efficiency.

## 1 Introduction

**Figure 1:**
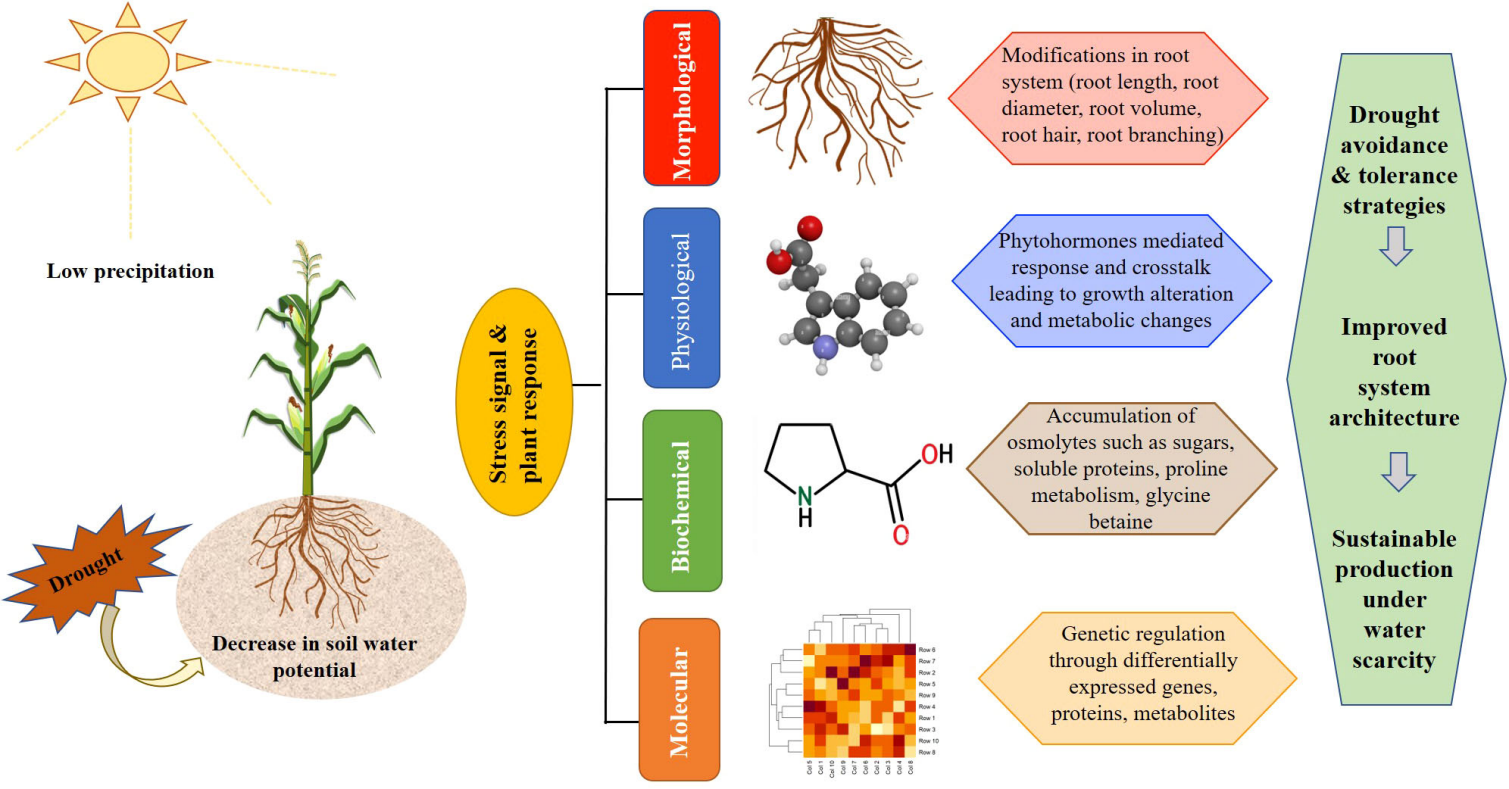
Various response mechanisms triggered in plants after perception of drought stress stimuli by plant roots.

Abiotic stresses, such as extreme temperatures, salinity, and drought are increasingly threatening global agricultural productivity, particularly in essential food crops (Harrison, 2021; Bai et al., 2022; Salehi et al., 2023; Rahnama et al., 2024). Drought is believed to have affected around 75% of harvestable land globally, resulting in cumulative production losses estimated at 166 billion U.S. dollars (Kim et al., 2019). Drought exerts a detrimental effect on plant diversity across multiple biological scales. This manifests through morphological impairments (reduced leaf area and stunted elongation), physiological disruptions (decreased stomatal conductance and leaf water potential), and biochemical shifts (ROS accumulation and proline synthesis) (Kaur and Behl, 2010; Kaur et al., 2017; Gupta et al., 2020). Additionally, anatomical alterations such as xylem cavitation further compromise overall growth and development (Kaur et al., 2011). Identifying and developing superior germplasm with enhanced water use efficiency is crucial for minimizing yield losses and attaining global food stability (Manju et al., 2019; 2023; Kaur et al., 2024). Plants are sessile beings that need to adapt to variable environmental cues through phenotypic plasticity involving extrinsic and intrinsic mechanisms that can be broadly categorized into drought escape, drought tolerance, drought recovery, and drought avoidance. Key root traits and adaptive mechanisms to cope with drought stress at different structural and functional organization in plants are summarized in **Table 1**. Among these, drought avoidance is the primary mechanism achieved through several metabolic and structural adaptive traits involving minimizing water loss and optimizing water uptake. These adaptive traits refer to complex processes governed by intricate regulatory networks involving numerous genes that, in turn, trigger varied morpho-physiological and biochemical adaptations such as increased root growth, reduced leaf area, accumulation of osmoprotectant, ABA-mediated regulation of stomatal aperture, and cytokinin-induced delayed leaf senescence (**Figure 1**). Enhanced or improved root growth remains the key modus for maintaining cellular water and nutrient equilibrium under moisture-deficit conditions. The degree of interaction between the root and its perirhizomal environment relies on its spatial arrangement and structure at the cellular to whole-plant level. To ensure maximum resource sequestering with the least metabolic cost, plants alter their Root System Architecture (RSA) in response to drought stress (Afonso et al., 2025). RSA refers to the spatial distribution of all root parts, including the root axis, number, seminal and lateral roots, density, and length of root hairs (Lynch, 1995; de Dorlodot et al., 2007; Wang et al., 2024). In addition to structural modifications, roots also govern numerous metabolic activities in the shoot, leaves, and other aerial parts of the plant upon sensing moisture heterogeneity in the soil (Maurel and Nacry, 2020). For instance, several reports fortify that photosynthetic assimilates are mobilized to roots rather than shoots in a water-scarce environment. This strategic carbon reallocation provides the energetic foundation required for the dynamic spatial remodeling of the root system. There is increasing acceptance that optimizing RSA and its interaction with the environment will play a key role in enhancing crop productivity, especially under input-starved conditions (Silim et al., 1993; Price et al., 2002b; Ober et al., 2005; Kaur et al., 2020; Manju et al., 2019; 2023; Sonia et al., 2023; Rahnama et al., 2024; Singh et al., 2024).

**Table 1:** Key root traits and adaptive mechanisms to cope with drought stress at different structural and functional organization.

Level	Key adaptive mechanisms	Main functional benefits	Specific examples/markers
Morphological	Root architecture modification	Maximizes water foraging	Increased root-to-shoot ratio
	Leaf area reduction	Minimizes transpiration	Early leaf senescence
	Dynamic leaf movement	Reduces light intercept and heat load	Reversible leaf rolling or folding
	Altered biomass allocation	Optimizes resource use for survival.	Shortened life cycle
Physiological	Regulation of stomatal aperture	Immediate regulation of water vapor exit	ABA-mediated closure signals
	Turgor maintenance	Sustains growth during low water potential	Active osmotic adjustment
	Modulation of photosynthesis (Pn)	Protects the photosynthetic apparatus	Non-photochemical quenching
	Stem/Leaf hydraulic conductivity	Prevents xylem cavitation and embolism	High Water Use Efficiency (WUE)
Biochemical	Biosynthesis of osmoprotectants	Stabilizes membranes and biomolecules.	Proline, Betaine, and Trehalose
	Antioxidant upregulation	Clears harmful oxidative molecules (ROS)	Enzymes like SOD, CAT, and APX
	Synthesis of stress-responsive proteins	Prevents protein denaturation	Synthesis of stress-responsive proteins: LEA and Heat Shock Proteins (HSPs)
	Metabolic reconfiguration	Shifts resources to stress-defense pathways	Increased phenolic compounds
Anatomical	Dermal barrier reinforcement	Forms a hydrophobic barrier to water loss	Thickened epicuticular wax/cuticle
	Stomatal architecture	Increases boundary layer resistance	Sunken stomata or dense trichomes
	Vascular structural change	Provides mechanical strength against collapse	Small, thick-walled xylem vessels
	Development of specialized tissues	Enhanced internal water storage/transport	Formation of aerenchyma or succulence

Evolutionarily, plants have been classified as monocotyledonous and dicotyledonous, possessing distinct root morphologies. Monocots have a fibrous root system, while dicots possess a tap root system. While monocots and dicots possess fundamentally different root systems, a shared characteristic is their tendency to branch profusely and alter architecture in response to the external environment to access resources (Morris et al., 2017). Therefore, understanding root architecture and the underlying molecular mechanisms governing its adaptation must be deciphered to identify and develop resilient genotypes. However, challenges in evaluating plant root systems are coupled with poorly understood genetic control. Firstly, most methodologies employed for assessing RSA are destructive sampling techniques, often resulting in the loss of finer-scale root features (fine lateral roots and root hair) (Bucksch et al., 2014; Wang et al., 2024). Secondly, destructive root phenotyping approaches are labour-intensive, time-consuming, and involve a high rate of root loss. Thirdly, the optimal root systems for crop growth vary vastly and are largely unknown due to the variations in environmental conditions. This environmental heterogeneity necessitates the deployment of high-throughput, precision technologies capable of capturing dynamic root responses across diverse soil-moisture profiles. Therefore, *in situ* non-invasive root phenotyping approaches are necessary for deciphering RSA with its environmental dynamics. The present review comprehensively elaborates on various aspects and progressions made in the realm of *ex-situ* (conventional) and *in-situ* root phenotyping (3D representation) approaches, with a brief insight into imaging systems and processing. Here, we summarized functionally significant root architectural traits in relation to root morphology, alongside the underlying molecular and physiological processes that govern them. Crucially, these physiological processes, including phytohormonal crosstalk and water-potential signaling, serve as the primary regulatory drivers that translate environmental stress into specific architectural modifications. Understanding these links is essential for deciphering how RSA is dynamically regulated to sustain plant performance under drought stress. Given that genetic factors largely govern root anatomy and architecture in conjunction with extrinsic or environmental factors, numerous QTLs, genes, and transcription factors governing RSA under drought stress have been elucidated. Further enhancements in RSA have been achieved efficiently through various genetic engineering and genome editing approaches lately. Finally, we have outlined the key roles of QTLs, genes, and phytohormones in modulating the response of RSA towards drought tolerance. These genetic and hormonal regulators control the partitioning of metabolic resources, balancing the energy cost of expansive root proliferation against the maintenance of shoot biomass and yield stability. Such insights are currently being explored through precision breeding and genome editing to optimize crop performance under water-limited conditions.

### 1.1 Precision phenotyping of RSA

Studying root systems has traditionally been hindered by their subterranean location. Nonetheless, recent technological breakthroughs have yielded diverse methods to characterize root architecture, which can be broadly classified into destructive and non-destructive approaches.

### 1.2 Destructive approaches

Destructive or invasive root phenotyping involves the physical removal or disruption of the plant and its subterranean system to analyze architectural and morphological traits. These procedures range from partial excavation, where localized sections of the root system are exposed for observation, to complete removal from the growth substrate. Such methods are inherently restrictive, as they typically result in the termination of the plant’s life cycle and the loss of fine-scale features like lateral roots and root hairs. The characterization of RSA under controlled environments often relies on substituting soil with alternative media to enhance image acquisition and data throughput. Various High-throughput RSA phenotyping platforms have been established, utilizing semi-solid gels to maintain 3D root topology (Nagel et al., 2009) and paper-based or fabric-based 2D rhizotrons that constrain root growth to a single plane for rapid quantification (Hund et al., 2009; Chen et al., 2011; Le Marié et al., 2016; Rahnama et al., 2011). Additionally, hydroponic cultures (Pace et al., 2014) and pouches (Adu et al., 2014) offer precise control over nutrient availability for rapid screening of root traits in a non-soil environment (Pace et al., 2014).

**Figure 2:**
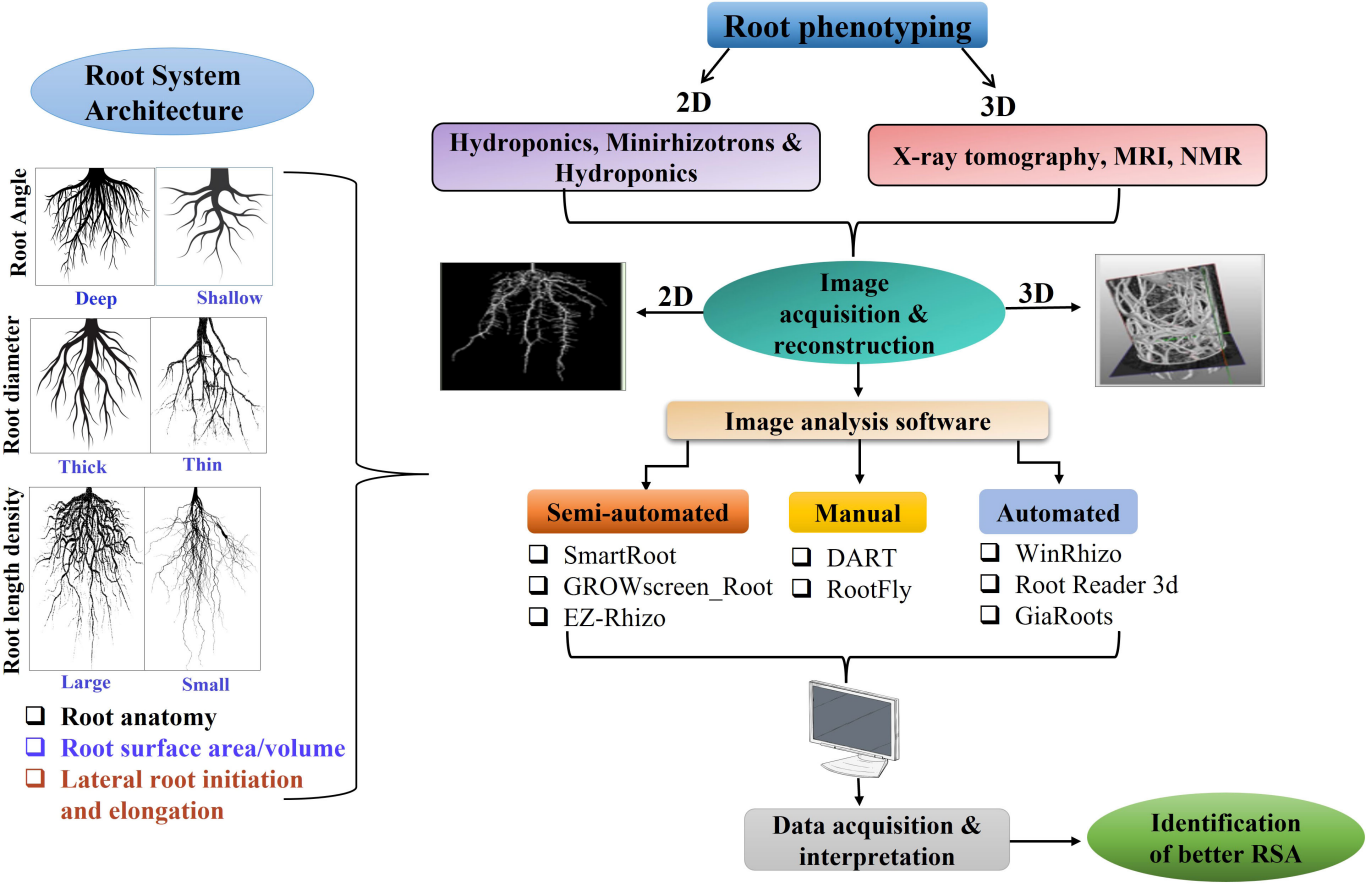
Phenotyping techniques and image analyses for identification of better root system architecture (RSA).

Such systems offer convenient operation and reduced environmental noise, resulting in greater efficiency and a standardized micro-environment. Additionally, these methods efficiently derive 2D/3D root architecture parameters by imaging roots from multiple view angles and subsequent analyses (**Figure 2**). These technologies allow for the precise quantification of critical spatial traits, including root growth angle, maximum vertical depth, total root volume, and convex hull area. By integrating these high-resolution metrics, researchers can construct a holistic representation of the root system’s spatial exploration within the soil profile. However, the constrained 2D growth and artificial composition of the growth medium limit the applicability of these methods to the agronomic utility that is confined to the seedling stage, which may not always be representative of the mature plant (Watt et al., 2013). Furthermore, RSA is influenced by a complex array of edaphic factors. Physical constraints such as mechanical resistance and bulk density, alongside chemical variables like pH, nutrient availability, temperature, oxygen availability, and microbial composition, influence root development. Exploiting transparent or opaque soil offers a promising approach that balances visibility and soil-like simulative mechanical resistance for root studies (Downie et al., 2012). Given the challenges of accurately simulating these soil conditions in artificial systems, field-based phenotyping is crucial for comprehensive root system evaluation.

Field-based RSA phenotyping relies on diverse methods, including soil-filled rhizo-boxes or sand rhizotrons (Thaler and Pagès, 1995; Giulani et al., 2005; Devienne-Barret et al., 2006), soil-filled transparent glass rhizotrons (Nagel et al., 2009; Ytting et al., 2014), flat cartridges (Nagel et al., 2012), and trench excavation (Bucksch et al., 2014), particularly in *Arabidopsis*, rice, and maize. The conventional approach for soil-based RSA phenotyping involves coring or shovelomics excavation of soil blocks, followed by careful root extraction without damaging finer roots, and finally washing of roots for detailed analysis (Lynch, 2011; Trachsel et al., 2010; Iyer-Pascuzzi et al., 2010; Pierret et al., 2013; Rahnama et al., 2019; 2024). Although extraction and cleaning roots from soil inevitably alter their 3D topology, this approach has been extensively employed in root phenotypic studies. Despite limitations in capturing the full three-dimensional structure, these systems provide substantial information regarding root quantity, length, and overall size (Pierret et al., 2013; Kumar et al., 2014). Transparent glass rhizotrons provide a simulated 3D view of root growth and development, specifically for those root portions interacting with the transparent surfaces. The lysimetric system is another approach that consists of a set of long and large PVC tubes filled with several cubic meters of soil encapsulated with an arrangement to mimic field conditions and monitor water use (Vadez et al., 2008; 2013; Madaan et al., 2018). Nevertheless, the soil-based root phenotyping approach is time and labour-intensive. Additional challenges include low heritability, soil heterogeneity, and manual analysis being prone to subjective errors, particularly for quantifying minuscule root traits.

### 1.3 Non-destructive approach

High-throughput phenotyping provides a non-destructive method for assessing root traits and their correlations with crop performance in diverse environments. By avoiding destructive sampling, non-invasive root phenotyping enables researchers to optically analyse root traits for growth, development, and activity in its natural environment. Further, non-invasive root phenotyping can assist in selecting promising root trait phenotypes with superior WUE and yield-related attributes. The selection of phenotyping methods must be optimized according to the biological scale of the plant, the physical properties of the soil, and the specific architectural traits under investigation. The choice of phenotyping platforms is crucially governed by imaging resolution and inherent architectural differences between monocots and dicots. For the Poaceae family, the structural complexity of multiaxial root systems comprising embryonic seminal roots and post-embryonic nodal roots necessitates high-resolution modalities. In species such as rice and barley, X-ray Computed Tomography (CT) and Magnetic Resonance Imaging (MRI) are used to resolve microscopic lateral branching and root hair zones (Iyer-Pascuzzi et al., 2010; van Dusschoten et al., 2015). In contrast, the expansive taproot systems of dicots like canola (*Brassica rapa*) are effectively monitored using 2D rhizotrons or gel-based systems to track primary root elongation (Adu et al., 2014). For larger cereal crops, such as maize and wheat, where deep-rooting and crown architecture are key yield drivers, specialized platforms like RhizoVision and Shovelomics are employed to quantify nodal root angles (Lynch, 2019; Seethepalli et al., 2020). For high-biomass or storage roots, such as those in cassava, Ground Penetrating Radar (GPR) is preferred to capture coarse root bulking rates (Delgado et al., 2017). Ultimately, the optimization of the signal-to-noise ratio must be calibrated to the biological scale of the species, ensuring that the system’s spatial resolution matches the specific root diameter and growth habit of the plant under investigation (Wasson et al., 2020). Minirhizotrons are an *in-situ* analysis of root dynamics, in which transparent tubes are inserted into the soil equipped with specialized cameras that allow for undisturbed, high-resolution imaging of root growth and behavior at the soil-tube interface (Smit et al., 2000). Despite the availability of multiple rhizotron designs, their implementation is often hindered by high cost and operational complexity, limiting widespread adoption. Additionally, image acquisition is confined to white light and usually necessitates human intervention for tracing roots. However, independent groups have made several structural enhancements. For instance, implementing a Convolutional Neural Network (CNN) as a root detector for minirhizotron images of variable crop systems and environments, and utilizing a Robotic Minirhizotron System (RMR), has provided replicable results (Peters et al., 2023; Nair et al., 2023). Likewise, the integration of SoilCam has enabled a 360° panoramic view with multiple spectral imaging of roots in real-time (Rahman et al., 2020). Despite advanced capabilities, 2D-based root phenotyping techniques pose several limitations owing to growth, environmental, and technical hindrances. 2D images cannot fully capture the three-dimensional structure of roots, particularly their angles and orientations, which affect the analysis of root growth patterns. Further, 2D images do not provide direct information about root volume and density, as denser root systems tend to overlap, hindering accurate segmentation and analysis. Although 2D root phenotyping has contributed significantly to our understanding of root morphology, its inherent drawbacks have prompted the advancement of 3D imaging and analysis technologies to capture the intricate details of RSA. Several 3D imaging methods permit *in situ* visualization of root systems as they grow in natural soil, preserving the temperature gradient, topology, and composition attributes. The methods have been successfully implemented for the assessment of yield traits in crops under drought conditions (Wasaya et al., 2018). X-ray computed tomography (CT) has been extensively adopted for the soil-based three-dimensional configuration of roots (Gregory et al., 2003). Although desirable, limitations of this method include potential loss of detail due to lower resolution, challenges in analyzing complex 3D root systems, low data processing speed, and the requirement for immobile, costly equipment (Mairhofer et al., 2013). An alternative imaging strategy is magnetic resonance imaging (MRI) (Rascher et al., 2011; Metzner et al., 2014). Recently, MRI-based root phenotyping of 288 wheat seedlings has been reported (Pflugfelder et al., 2022) and designated as medium-throughput phenotyping. Integrating Magnetic Resonance Imaging (MRI) with Positron Emission Tomography (PET) facilitates the imaging of photosynthetically acquired carbon in the root system (Jahnke et al., 2009; Nagel et al., 2009). However, the presence of ferromagnetic soil components in iron-rich soil hinders the absolute analysis of root morphology (Flavel et al., 2012). Ground-penetrating radar is an alternate non-invasive geophysical instrument that employs electromagnetic waves to sense features below the surface, dependent upon the physical and dielectric attributes of materials. GPR distinguishes coarse roots from the surrounding soil due to their contrasting dielectric permittivity, registering them as hyperbolic reflections that these structures generate in the radar datasets (Lombardi et al., 2021). Although GPR technique has been employed in assessing root biomass, specifically using rhizosphere imaging (Lorenzo et al., 2010), the technique requires further refinement to achieve broad applicability in physiological and genetic investigations. Root Electrical Capacitance (REC) represents an alternative 3D phenotyping technique that establishes root structure analysis of low-frequency alternating current running between the plant stem and the surrounding soil medium. REC has been widely applied in RSA-based studies in maize, soybean, wheat, and some grassland species (Cseresnyés et al., 2020). In a recent advance, Rayleigh scattering-based fiber optic sensors (FOS) have been deployed to observe soil’s physical properties (such as strain, temperature, and vibration), thereby defining the RSA in real-time (Weihs et al., 2024). Continuous refinements are applied to these techniques and their utility for large-scale root phenotyping applications is due for a thorough investigation. Even as they continue to evolve, these approaches present unsurpassed capacity for the kinetics of root expansion within the rhizosphere.

## 2 Modeling and simulation

Following the acquisition of multiple images depicting the root system’s morphology, the primary task becomes transforming these visual representations into quantifiable information. A primary impediment to the automatized quantification of lateral root structure is distinguishing overlapping root networks, a problem particularly noticeable in fully grown plants propagated in both two-dimensional and three-dimensional systems. The problem is further aggravated in mature roots adhered to root particles, thereby compromising image quality and subsequent data analysis. Although, complex traits like total root area and length are easily quantified from images without manual input, as they don’t require identifying specific root sections. Addressing these requirements, a diverse array of trait-specific software exhibiting varying success rates has been developed. A comprehensive summary of diverse laboratory-to-field methodologies (**Table 2**) and the associated software for RSA phenotyping is provided in **Table 3**. These packages primarily function by generating a root hierarchy based on their morphological features, physiological roles, anatomical properties, spatial configuration, and growth dynamics. Although several packages can process images autonomously, numerous systems still require human intervention for the rectification of errors within the image processing. In addition, the majority of the software employs a skeletonization method incorporating both the topological structure and the concept of local object symmetries found within the roots. Many of the quantitative traits involving root numbers, diameters, lengths, and the number of root tips have been processed for image-based analysis using Image J (Schneider et al., 2012), WinRHIZO™, EZ-Rhizo (Armengaud et al., 2009), GROWSCREEN_ROOT (Nagel et al., 2012), IJ_Rhizo (Pierret et al., 2013), SmartRoot (Lobet et al., 2011), DART (Le Bot et al., 2010) RootTrace (Clark et al., 2013), RootNav (Pound et al., 2013)and Root System Analyzer (Leitner et al., 2014). Soil particles on roots can hinder the efficacy of tools like WinRHIZO™ and EZ-Rhizo, leading to inaccurate root measurements, such as root tip counts (Kumar et al., 2014). Accurately distinguishing primary and lateral roots is crucial for measuring how different root parts respond to varied stresses. Conventional software applications like SmartRoot, EZ-Rhizo, GROWSCREEN_ROOT, and Root System Analyzer are semi-autonomous but widely used for assessing growth kinetics and branching angles. However, ARIA (Pace et al., 2014), RTipC (Kumar et al., 2014), RootNav 2.0 (Yasrab et al., 2019), WinRhizo, Root Reader 3D (Clark et al., 2011), GiaRoots (Galkovskyi et al., 2012), and DIRT/3D (Liu et al., 2021) have been extensively used for high-throughput 3D autonomous extraction of complex RSA phenotyping, particularly locating first and second-order root tips without user interaction. Recent advancements encompass various root phenotyping models, including 3D, 4D, allometric, R-SWMS, and response surface models that can quantify changes in RSA resulting from external environmental stimuli. Several listed software packages offer pre-configured extensions or allow customization through open-source code. Besides improvement in image segmentation and reconstruction, the ultimate phenotyping systems should be flexible and fully automated to accelerate processing and increase accuracy.

**Table 2:** Various techniques for minimal to non-invasive analysis of root morphometric and growth dynamic parameters.

Techniques/Approaches	Parameters/Traits	Applications	Remarks	References
2D and 3D approaches applicable for root studies in the field/lab				
2D imaging of roots grown in germination paper rolls or pouches.	Root length and topology, root architecture, growth dynamics	Suitable for laboratory study	Affordable, easy, and non-destructive visualization of RSA, but limited information is provided, not high throughput	Trachsel et al., 2009; Hetz et al., 1996; Bengough et al., 2004; Hund et al, 2009; Fang et al., 2009; Chen et al., 2011
2D imaging of roots in hydroponic cultures	Root elongation rate, density, surface area, number, and length, root angle, growth dynamics	Suitable for laboratory study	Optical visualization of the entire root system, non-destructive, but a different kind of root growth due to lack of mechanical impedance, a permanent water supply	Jones, 1982; Tuberosa et al., 2002
Gel-based systems (agar/gelatin gums); imaging by flatbed scanners, digital cameras, or lasers (3D scanning)	Root elongation rate, density, surface area, number, and length.	Suitable for laboratory study	Easy and non-destructive visualization of RSA, but the roots are generally exposed to light. Additionally, there is the potential for hypoxia.	Nagel et al., 2006; French et al., 2009; Fang et al., 2009; Iyer-Pascuzzi et al., 2010; Clark et al., 2011; Topp et al., 2013; Ribeiro et al., 2014
Soil-filled rhizoboxes	Root morphometric and growth dynamic traits	Suitable for laboratory study	larger monitoring area; problems for automatic data evaluation	Thaler and Pagès, 1995; Giulani et al., 2005; Devienne-Barret et al., 2006; Watt et al., 2006
Mini-rhizotrons; 2D imaging and analysis of root growth from a time series of images using software or integrated platforms	Root elongation rate, density, surface area, number, and length at different soil depths, estimating root production and turnover, exploring kinematic or morphometric root growth	Applicable to field studies	minimally invasive method; space may be created around the soil-tube interface that could influence root growth if the tubes are not installed properly, a limited part of the RSA under observation	Gregory, 1979; Thorup-Kristensen, 1993; Smit et al., 2000; Johnson et al., 2001; Williams and Weil, 2004; Chapman et al., 2011
X-ray tomography	Morphometric parameters in 3D	Suitable for laboratory study	3D root analyses, enabled, a costly affair, require long scan times	Heeraman et al., 1997; Gregory et al., 2003; Flavel et al., 2012; Pierret et al., 2003; Tracy et al., 2010; Zhu et al., 2011
Magnetic resonance imagers (MRI)	Morphometric parameters in 3D, water content	Suitable for laboratory study	3D root analyses enabled; Removal of ferromagnetic elements in soil required, a costly affair, requires long scan times	Menzel et al., 2007; Jahnke et al., 2009; Rascher et al., 2011
Biospeckle imaging	Root tissue and growth	Suitable for laboratory study	Analyze the changes in root tissue heterogeneity	Ribeiro et al., 2014
Ground-penetrating radar and electrical resistivity tomography	Root shape, distribution, and volume of the root system in 2D and 3D.	Applicable for both laboratory and field studies	Imaging of the rhizosphere	Zenone et al., 2008
Soil-filled tubes	Depth of soil layer, mean soil density, gravimetric water content	Applicable to field studies	Root tip, structure, and function: Measure the root depth of younger plants	Nagel et al., 2009; Ytting et al., 2014

**Table 3:** Comparative overview of digital phenotyping platforms for quantifying multidimensional RSA and physiological indices.

Imaging system/Software	Parameters/Traits	Applications	Remarks	References
SmartRoot	Root morphology, geometry, topology, and global parameters	Semi-automated software	Not used for global parameters	Lobet et al., 2011
EZ-Rhizo	Morphology, geometry, topology, and global parameters of the root	Semi-automated software	Detects 0.1-mm lateral roots on the main root, but is limited to simple root systems and requires extensive manual selection of image evaluation parameters	Armengaud et al., 2009
GROWScreen- Root	Root morphology, geometry, topology, and global parameters	Semi-automated	Also been applied to image time series in rhizotrons	Mühlich et al., 2008; Nagel et al., 2006; 2012
WinRhizo	Root length and topology	Automated	Analyzes washed roots	Arsenault et al., 1995
Root Reader 3D	Root morphology, geometry, topology, and global parameters	Automated	Image acquisition with a 3D laser scanner or camera, and rotation of the target	Fang et al., 2009; Clark et al., 2011
GiaRoots	Global morpho-geometric parameters	Automated	Image acquisition with a visible camera and rotation of targets	Galkovskyi et al., 2012; Iyer-Pascuzzi et al., 2010
Intrinsic root coordinate system (iRoCS)	Root diameter, localization, and volume 3D reconstruction	Automated/Semiautomated	Enables 3D resolution and detects nuclei or segments of cells, and the nuclei/cells are grouped into the root’s tissue layer	Schmidt et al., 2014
Root traking (RooTrak)	3D reconstruction	Semiautomated	Utilize visual-tracking techniques and different models of root material appearance in μCT data. Visualize fibrous and herring-bone root systems of monocot and dicot species, respectively.	Mairhofer et al., 2012
Automatic Root Image Analysis (ARIA)	Root shape, count length, and convex hull.	Automated	Convert root images into graphs	Pace et al., 2014
Analyzer for root tip tracks (ARTT)	Velocity profile, orientation, and localization of the root	Automated	2D kinematic detector software that detects and tracks root tips and specifies physical quantities such as trajectory, displacement, velocity, direction, and orientation.	Russino et al., 2013
Busch-lab Root Analysis Toolchain (BRAT)	Multiple basic traits of the primary root (e. g. root length, root width, root topology, and growth direction)	Automated	Enables high-throughput phenotyping of early root growth and development.	Slovak et al., 2014
Grow Map-Root	Root velocity profile	Automated	Visualize and quantify the growth rates of root tip segments using spatio-temporal dynamics.	Walter et al., 2002
Growth explorer	Root velocity profile	Automated	Identify the details of developmental and growth parameters	Basu and Pal, 2012
GiA Roots	Root depth, length, number of branches, surface, volume, convex hull	Automated	Identify and characterize root system architecture	Galkovskyi et al., 2012
IJ-Rhizo	Root length and diameter	Automated	Measures scanned images of root samples with accuracy.	Pierret et al., 2013
Data *Analysis* of *Root* Tracings (DART)	Root length, topology, and insertion	Manual	Identify roots across time series	Le Bot et al., 2010
KineRoot	Root growth and gravitropism	Automated	Identify spatio-temporal patterns of local root growth and curvature data overall several hours.	Basu et al., 2007
Root and nodule quantification software (RNQS)	Root length and nodules	Automated	Visualize the roots of seedlings using 2D scans	Remmler et al., 2014
Root System Analyzer	Root length, diameter, insertion angle	Automated	Track primary and lateral roots in a fully automated way	Leitner et al., 2014
Root Tip Detection	Count, diameter	Automated	Detect root phenotyping properties either by 2D flatbed scanning or by 3D digital camera imaging	Kumar et al., 2014
RootFlowRT	Root growth and velocity profile	Automated	Measure root growth at high spatial and temporal resolution	van der Weele et al., 2003
RootFly	Root length, diameter, and colour	Manual	Root image analysis using 2D scans	Zeng et al., 2008
RootNav and RootNav 2.0	Root length, count, insertion, insertion angle, and convex-hull.	Semi-automated	Quantify root system architecture using the EM clustering algorithm	Pound et al., 2013; Yasrab et al., 2019
RootReader2D	Root length, depth, topology, and number of branches	Semi-automated	Quantify the roots of hydroponically grown plants	Clark et al., 2013
RootScape	Root shape	Semi-automated	Quantify root and integrative phenotyping of the RSA in *Arabidopsis*	Ristova et al., 2013
RootTip Trace	Root length	Semi-automated	Detect the hormone signaling pathway using high resolution spatio-temporal transcriptional map	Geng et al., 2013
RootTrace	Root length, number of branches, and curvature	Automated	Measure a variety of growth parameters on several roots throughout a long-time series	French et al., 2009
RhizoNet	Robust calculation of biomass and growth kinetics across time-series data	AI-based	Designed to automate root segmentation in hydroponic systems with high precision	Sordo et al., 2024
RootPainter	Facilitates the extraction of root length density, nodule distribution.	Graphical User Interface (GUI)	Overcomes limitations of traditional imaging by isolating RSA from complex backgrounds.	Smith et al., 2022
RootXplorer	Penetrability & Spatio-temporal growth of roots	Automated	Quantifies “penetrability” and 3D growth kinetics, which conventional 2D scanners cannot capture	Bello et al., 2025
ORS Dragonfly	Volumetric surface area, network orientation, skeleton length, and soil-root contact	Semi to Fully Automated	Uses AI to segment roots from 3D X-ray CT scans	Duncan et al., 2022
RooTrak & RooTh	Total root volume, 3D tortuosity, and branching hierarchy in soil.	Fully Automated	3D root skeletonization from X-ray Micro-CT data	Mairhofer et al., 2012
RootReader3D	Convex hull volume, 3D root tip distribution, and total network length.	Semi-Automated	Reconstructs 3D models from 2D rotations.	Clark et al., 2011
RhizoVision	Root length, average diameter, volume, fineness, and number of root tips	Automated	Superior speed for large populations; automated “broken root” logic for discontinuous segments	Seethepalli et al., 2021
SmartRoot	Insertion angles, individual root growth rates, and precise topological linkages	Automated	Employs geometric paths rather than pixels, resolving overlaps and enabling longitudinal analysis	Lobet et al., 2011
MARSHAL	R-based tool quantifies hydraulic conductivity, water uptake efficiency, and transpiration proxies	Automated	Goes beyond structure to simulate water movement, providing direct proof of drought efficiency	Meunier et al., 2020

### 2.1 Artificial intelligence assisted root phenotyping

AI can revolutionize the field of root phenotyping. Conventional methods for root analysis are labour and time-intensive and are primarily destructive approaches. By implementing machine learning (ML) and computer vision techniques, AI is significantly streamlining and enhancing root analysis protocols, offering crucial perspectives on root architecture, dynamic development, and resilience against extreme environmental cues. Fundamentally, AI relies on the principle of machine learning, a methodology that enables systems to learn patterns and execute judgment without prescriptive coding. Notably, ML’s major advantage involves its intrinsic ability to generalize underlying trends following intensive data assimilation. Further developing this capability, Deep Learning (DL) constitutes a specialized subset of machine learning, relying on representation learning to build models of considerable sophistication. ML’s effectiveness stems from its capability to generalize knowledge from assimilated data, while deep learning (DL), a specialized subset, utilizes representation learning for constructing intricate conceptual models.

Deep Learning, consequently, encompasses the acquisition of hierarchical feature layers like edges, localized motifs, and individual object components, holistically representing image data. The model secures these features utilizing general-purpose learning mechanisms that function autonomously, free from human input (LeCun et al., 2015). Comprising DL convolutional neural networks (CNNs), and recurrent neural networks (RNNs) are prominent subclasses. Designed for 2D image data, Convolutional Neural Networks (CNNs) are optimized for tasks centered on spatial relationships. However, Recurrent Neural Networks (RNNs), analyze sequential data, making them appropriate for inputs such as time-series data, streams of video, and even certain image types. Conversely, AI-based high-resolution analyses significantly improve downstream segmentation and data interpretation steps, particularly the use of computer vision (CV) and deep learning (DL) has rapidly gained distinction as the conventional approach in plant phenomics research. Numerous AI-driven tools are now largely being employed for RSA assessment. CNN-based Caffe DL library has been used for assessing the presence or absence of root tips in an image with an accuracy of 98.4% and overcoming overfitting issues through the random deactivation of fully connected neurons during each iteration. Several graphical user interface (GUI) DL segmentation and annotation tools, such as RootPainter segmentation and RhizoVision Explorer, have been widely used for branch-style, taproot-style, and taproot-branch roots quantification (Xu et al., 2022). Grayscale images of greenhouse-grown pea cultivars were analyzed using WinRhizo 2012b Pro to extract root features such as diameter, length, and surface area. To mitigate low signal-to-noise ratios in root imagery, hybrid machine learning frameworks—integrating radial basis function Support Vector Machines (SVM) and Random Forest (RF) classifiers have been explored, achieving up to 86% accuracy in feature extraction (Zhao et al., 2016; 2017). Recent advancements, such as RootNav 2.0, leverage multitask Convolutional Neural Networks (CNNs) to automate both image segmentation and root order assignment. This deep learning approach facilitates robust architectural quantification across diverse seedling morphologies, including Arabidopsis, rapeseed, and wheat, using standard RGB input (Yasrab et al., 2019). Further, the consortium of SVM, KNN, and linear discriminant analysis (LDA) algorithms has been used for the detection of root decay in Wheat (Shu et al., 2021). These highlighted examples illustrate several major developments within RSA image analysis, driven by enhanced processing speed resulting from the evolution of accelerated graphics processing units (GPUs). Current efforts emphasize leveraging ML for full automation in segmentation and achieving root detection with greater efficiency and precision, accomplished via a diverse array of sensors. In this realm, SegNet and U-Net are the two popular convolutional neural network (CNN) architectures widely used for semantic segmentation tasks, including root phenotyping. Despite their shared characteristics, SegNet and U-Net are potent CNN frameworks for root phenotyping, exhibiting crucial distinctions in structure and overall output. Selecting one necessitates evaluating context-specific factors, including image dimension, complexity of root structures, and access to computing power. U-Net is frequently utilized in root phenotyping because its skip connections preserve high-resolution spatial features, allowing for the accurate segmentation of fine lateral roots. However, AI-driven analysis is currently constrained by the scarcity of expert-annotated ground-truth datasets and geometric occlusion from overlapping root structures. Furthermore, the algorithmic opacity of deep learning necessitates the integration of Explainable AI (XAI) to ensure the biological interpretability and cross-platform reproducibility of architectural traits. Overcoming these challenges will be crucial for unlocking the full potential of AI in advancing our understanding of plant root biology.

## 3 Root morphological adaptations for regulation of water acquisition

Several root traits, such as root length density, branching, root diameter, root cap, root hairs, root hydraulic conductance, and root hydro-stimulation, can significantly contribute to trait-targeted breeding to achieve sustainable yield and drought resistance. Major root traits of significant importance in leveraging water uptake have been summarized in **Supplementary Table 1**.

### 3.1 Deep root growth and root length density

Root length density (RLD) is a measure of the total length of roots per unit volume of soil, serving as an indicator of lateral root expansion. Water uptake is positively correlated with RLD up to a critical threshold and is largely governed by soil moisture (Siopongco et al., 2005). A higher root length density, characterized by deeper and more abundant root growth, is generally associated with improved drought tolerance (Gowda et al., 2011; Henry et al., 2011; Lynch, 2013; Rahnama et al., 2024; Wasson et al., 2012). Drought stress triggers a root system response, characterized by increased fibrous root production, decreased lateral root width, and a shift in root biomass (Smith and De Smet, 2012; Manju et al., 2023; Sonia et al., 2023; Singh et al., 2024; Afonso et al., 2025). Research suggests extensive root development is advantageous in regions reliant on seasonal rainfall. Thus, less RLD in shallow layers of soil and more RLD in medium/deep soil layers has been considered as an efficient breeding strategy in environments subjected to the availability of deep water later in the crop season (Wasson et al., 2012; Lynch, 2013). Also, deep water extraction would be more beneficial if it is available during the most critical water demand, as shards of evidence suggest for higher grain yield wheat (Kirkegaard et al., 2007; Manschadi et al., 2006), chickpea (Zaman-Allah et al., 2011; Kashiwagi et al., 2015), and pearl millet (Vadez et al., 2013). While deep and profuse root systems are valuable for water acquisition, they are not the sole determinants of a reliable water supply. For example, in a mapping population between the deep-rooted rice Azucena and a shallow-rooted Bala, the water-conserving shoot traits from Bala were more pronounced than the root traits from the Azucena allele (Price et al., 2002a). Concisely, although roots are potentially important for the uptake of moisture in plants, they may or may not contribute to drought adaptation in all stress conditions owing to: (i) RLD, deep or profuse rooting is not very beneficial for water extraction in shallow soils/in the soil where there is no water at depth, or under conditions of mild water stress; (ii) root growth is closely coordinated with shoot growth and eventually deeper rooting might lead to faster soil water depletion.

### 3.2 Root hairs

Root hairs are epidermal cell extensions covering around 77% of the surface area and are considered imperative for water and nutrient uptake (Gilroy and Jones, 2000; Emons and Ketelaar, 2009; Libault et al., 2010; Datta et al., 2011). Root hairs substantially increase root surface and thus have a key role in water absorption and thereby compensate for reduced root length in response to abiotic stressors, primarily drought, heat, and salinity (Wasson et al., 2012; Rahnama et al., 2011; 2019; 2024; Wang et al., 2024). Kato et al. (2013) correlated the water absorption with root hair length while investigating a root hair-less *Arabidopsis* line, NR23, implying that the amount of absorbed water is dependent upon the abundance of root hairs. It was also suggested that an increase in the number of lateral roots could not compensate for the loss of root hairs in NR23. Tanaka et al. (2014) also opined that a substantial amount of water was absorbed through root hairs, while other root epidermal cells could be responsible for the remaining uptake and absorption. Barley mutants lacking root hairs exhibited reduced water uptake despite having extensive root branching (Segal et al., 2008). In addition to increasing the root’s absorptive surface, root hairs can grow into minute pores and attach themselves to soil grains, leading to intercepting and sequestering immobile or sluggish nutrients, primarily soil-bound ferrous and phosphorous particles.

### 3.3 Root xylem vessels size, abundance, and hydraulic characteristics

Plants with long specific root lengths and thinner diameters can efficiently increase water uptake by expanding their contact area with the soil and enhance hydraulic conductivity by counteracting the xylem’s apoplastic barriers (Rieger and Litvin, 1999; Solari et al., 2006; Hernández et al., 2010). Further, larger xylem vessels and increased metaxylem numbers are better equipped to absorb water from deeper soil layers under drought stress, owing to improved hydraulic conductivity in soybean and olives (Prince et al., 2017; Tan et al., 2020). Accordingly, reduced root diameter has been advocated for increased acquisition of water under drought (Wasson et al., 2012). In rice, drought-tolerant cv. Dular was reported to have a smaller xylem diameter than drought-susceptible IR64 under water stress conditions (Henry et al., 2012). Also, the smaller diameter of metaxylem vessels contributes to increased resistance to cavitation (Guet et al., 2015). In wheat, the reduced metaxylem diameter of seminal roots resulted in enhanced yield attributes such as yield performance and grain yield under drought stress (Richards and Passioura, 1989). It has been reported that xerophytic plants have fine roots with smaller diameters and more SRL (Hernández et al., 2010; Henry et al., 2012). Moreover, the abundance and increased conductance of aquaporins, responsible for the regulation of the passage of water acquisition, are associated with increased root hydraulic conductivity and thus may lead to compensation for reduced surface area (Parent et al., 2009; Vandeleur et al., 2009; Laur and Hacke, 2013).

### 3.4 Root cap and hydrotropism

The root cap has sensory cells responsible for sensing the moisture gradient through a phenomenon known as hydrotropism (Miyazawa et al., 2008; Takahashi and Scott, 1993; Takahashi et al., 2003). Hydrotropism is important for controlling the growth orientation of roots, obtaining water, establishing a plant stand, and thus drought avoidance. However, the degree of orientation or bending of the roots varies with species. For example, de-tipped roots do not alter hydrotropism in rice (Nakajima et al., 2017) and cucumber (Fujii et al., 2018) seedlings. In contrast, de-tipped root caps in pea and maize block hydrotropism. The removal of this tissue eliminates the site of hydro-sensing and the subsequent generation of signal transducers, such as abscisic acid (ABA), which are required to induce the differential growth that drives root curvature (Eapen et al., 2005; Takahashi and Scott, 1993). *Arabidopsis* is a popular model for studying hydrotropism due to its high sensitivity (Eapen et al., 2005; Takahashi et al., 2009; Cassab et al., 2013; Miyazawa et al., 2009; Saucedo et al., 2012). *A*. *thaliana* mutants with impaired or altered hydrotropism were identified and named ‘mizu-kussei’ (miz), expressed primarily in root tips and hydathodes (Kobayashi et al., 2007). Interestingly, *MIZ1* domain homologs are not reported in animal or microbial genomes, suggesting their exclusive role in terrestrial plant species. Thus, evident that *MIZ1* plays a crucial role in sensing moisture gradients and root hydrotropic response, which might be a stress avoidance strategy to cope with drought, as evidenced in Kaur et al. (2020). Other hydrotropic mutants, nhr1 and ahr1, were also reported (Eapen et al., 2003; Saucedo et al., 2012**).** The nhr1 mutant did not display hydrotropism in the presence of water potential gradients but displayed ortho-gravitropism (Eapen et al., 2003). The site of water perception and growth regulation was determined to be located in the root cortex (Dietrich et al., 2017). Intriguingly, hydrotropism was found to be independent of polar auxin transport, as the inhibition of both influx and efflux carriers of auxin did not alter the hydrotropic response (Kaneyasu et al., 2007).

## 4 Determinants governing RSA

Comprehending the mechanisms regulating root architecture and hydropatterning is crucial for developing crops that exhibit enhanced resilience to abiotic stresses, especially drought. These include phytohormone-mediated regulation and genetic regulation of RSA.

### 4.1 Phytohormone-mediated regulation of RSA

**Figure 3:**
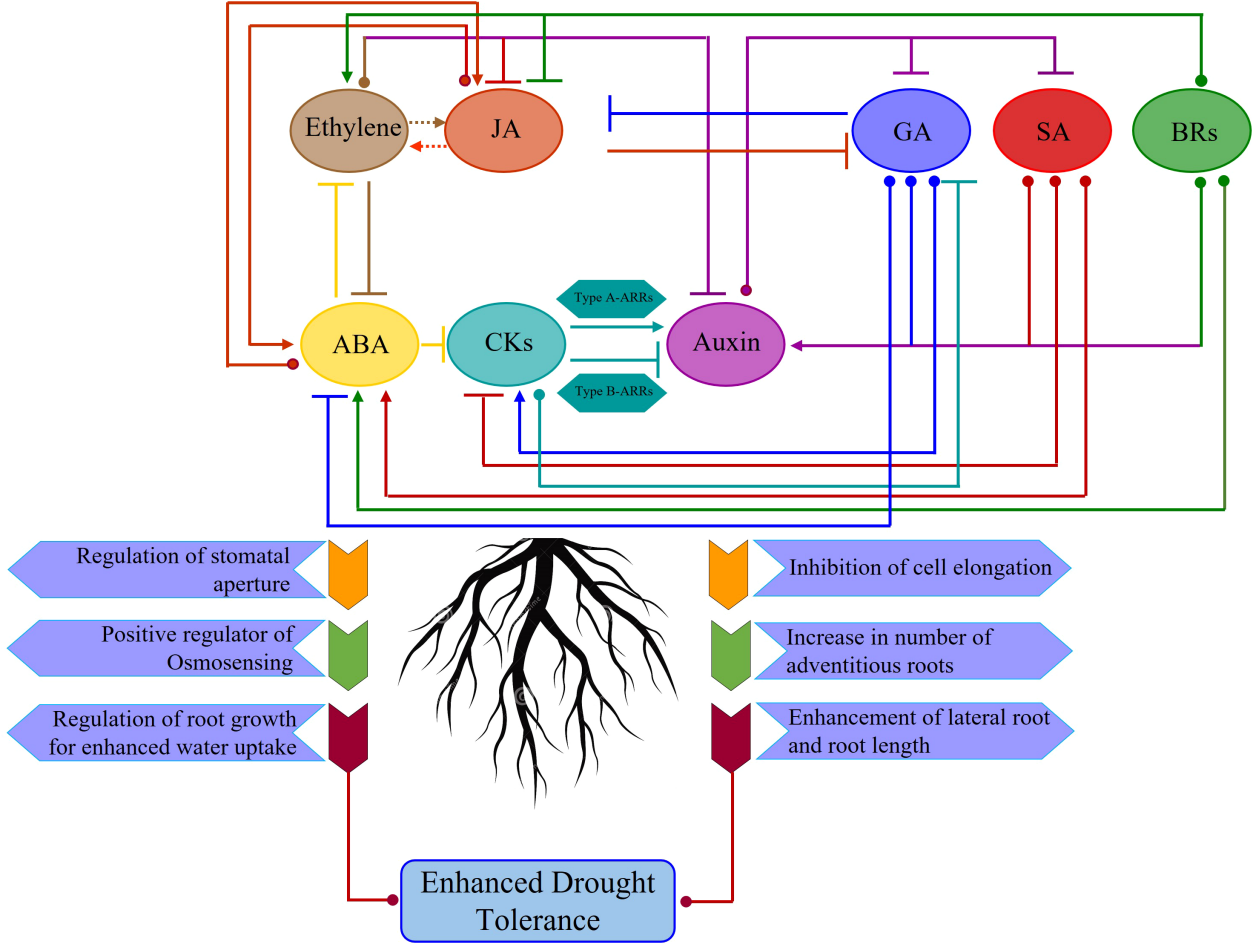
Hormonal interactions in regulating root architecture in response to drought stress. Arrowheads indicate a positive interaction, whereas a T end indicates an inhibitory effect. The central role played by phytohormone Abscisic acid (ABA) and Auxin in imparting drought tolerance. Phytohormone ABA has strong synergistic effects on Jasmonic acid (JA)-dependent defenses, while JA promotes ABA-mediated stomatal closure and leaf senescence, but not root growth. Dashed arrows indicate the mixed effects of ethylene (ET) on JA-dependent defenses. ABA and ET antagonize each other in numerous stress responses, but both induce leaf senescence. Gibberellic acid (GA), Salicylic acid (SA), and Brassinosteroids (BRs) positively regulate auxin signaling, thereby promoting root growth for higher water uptake and survival in response to osmotic stress. Cytokinin (CKs) positively regulates auxin signaling, thereby promoting primary root growth via Type A-ARRs at low Auxin concentration, while it negatively regulates auxin signaling via Type B-ARRs at higher auxin concentration.

Roots are the primary organs for perceiving and transmitting osmotic stress through osmosensors in response to abiotic stresses. The complex interplay of phytohormone homeostasis, signaling, and their interactions plays a crucial role in hydrotropic responses under favorable and adverse environmental conditions. Abiotic stresses, particularly drought stress, trigger a series of signal cascades involving the production, distribution, and signaling of phytohormones, including abscisic acid, auxin, cytokinin, ethylene, jasmonic acid, and brassinosteroids, impacting drought responsiveness in plants (**Figure 3**).

Abscisic acid (ABA) is the key stress hormone that has a synergistic effect on primary root elongation, lateral root development, and branching (Yadav et al., 2025). In roots, the interplay between ABA signaling and the modulation of auxin biosynthesis and transport is responsible for minimizing water loss and maximizing water acquisition (Lamers et al., 2020). Water deficit leads to an increase in the concentration of cellular ABA, which binds to PYR/PYL receptors. This leads to a decrease in auxin concentration and thereby inhibition of lateral root growth (Bloch et al., 2019). ABA has been shown to positively regulate hydrotropism and hydropatterning of RSA (Dietrich et al., 2017; Dinneny, 2019). Miao et al. (2021) demonstrated that under osmotic stresses, ABA promotes hydrotropism by inhibiting PP2C phosphatase activity, thus promoting apoplastic H^+^ efflux via H^+^-ATPase2.

Stress-induced ABA flux regulates different components of RSA in various plant species. In the model legume *Medicago truncatula*, ABA plays a crucial role in maintaining the function of the root meristem (Liang et al., 2007), while in *Arabidopsis*, it stimulates the quiescence of the undifferentiated cells at the root tip and inhibits stem cell differentiation in the primary root meristem (Zhang et al., 2010). Tomato mutants lacking ABA exhibited significantly limited RSA under drought stress conditions (Zhang et al., 2022). Furthermore, ABA accumulation was positively correlated with decreased matric potential and turgor pressure in root growth zones of maize (Spollen et al., 2000). The regulation of primary root ontogeny in maize, alongside the spatial differentiation of cells within the wheat root apex, is fundamentally governed by ABA-mediated signalling in response to drought stress (Kang et al., 2022).

Phytohormone auxin plays a pivotal role in regulating the signal response to osmotic stress, triggering the growth and development of primary and lateral roots. Auxin primarily governs root development, centrally affecting cell elongation, differentiation, apical dominance, and varied environmental tropisms (Iqbal et al., 2022). Extensive studies on the role of auxin signalling preceding lateral root primordia formation, translating to lateral root initiation, growth, and elongation, have been reported in Arabidopsis (De Smet et al., 2007; Möller et al., 2017; Van Norman et al., 2013). Auxin has been shown to promote root branching under drought stress in tobacco (Wang et al., 2017). A higher concentration gradient of auxin in root tips and its polar movement regulate root hair genesis and elongation. However, auxin-mediated hydrotropism varies among plant species. Auxin positively regulates hydrotropism in rice, cucumber, and pea (Nakajima et al., 2017), whereas it acts as an antagonist compound enhancing hydrotropic response in *Arabidopsis* (Chang et al., 2019). Cytokinin (CKs) and auxin are two pivotal phytohormones that regulate root growth and development. Although their outcomes are frequently conflicting, their intricate crosstalk exerts both antagonistic and synergistic effects on root growth and development. CKs are integral components in various physiological processes, including cell division, shoot growth, and delaying senescence. CKs influence RSA by altering root morphology and metabolic activities in response to limited water availability growth regimes (Pospíšilová et al., 2016). Further, CKs are found to regulate the elongation of primary roots and the initiation of lateral roots in *Arabidopsis*. However, CKs are also known to negatively inhibit RSA development. Lower endogenous CKs in roots correlate with improved root architecture, elevated lateral root proliferation, and enhanced nutrient assimilation (Nehnevajova et al., 2019). However, drought stress induces increased cellular CK levels, leading to better root growth in overexpression lines than in non-transformed WT plants (Xu et al., 2016).

Ethylene, a gaseous hormone, links environmental stimuli to plant developmental adaptations by modulating cellular growth in the root apical meristem. This regulation is achieved through a synergistic crosstalk where ethylene promotes auxin biosynthesis and its transport to the epidermis. This hormonal convergence triggers the transcriptional activation of root-hair-specific genes, thereby driving hair initiation and elongation. This coordinated response maximizes the root surface area for resource acquisition under stress (Vissenberg et al., 2020). Further, the exogenous application of NAA (synthetic auxin) and ACC (ethylene precursor) (Muday et al., 2012) reinstated root hair formation. The ethylene signal typically mediates RSA plasticity to confer tolerance during osmotic stressors. Ethylene biosynthesis, coupled with K^+^ transport, governs the Na^+^/K^+^ homeostasis, controlling cellular expansion and lateral root morphogenesis in response to osmotic stress (Osakabe et al., 2013). Ethylene and ABA frequently interact synergistically, wherein an elevated ABA concentration in roots suppresses ethylene production, which in turn facilitates increased primary root length plasticity (Kang et al., 2022).

Jasmonic acid (JA) significantly modulates root architecture by balancing primary and secondary growth. While JA acts as a potent inhibitor of primary root elongation and meristematic activity, it serves as a positive regulator of lateral root formation Chen et al., 2011; Gutierrez et al., 2012; Lakehal and Bellini, 2019; Cai et al., 2014). Additionally, the exogenous application of JA to the hydroponic root culture in tomatoes caused inhibition of primary root growth, root diameter, and bulged root tips. Most of the processes are often coordinated with auxin signaling. The convergence of these distinct signaling pathways into integrated hormonal networks is fundamental to orchestrating the plant’s adaptive response to drought. Rather than operating in isolation, these networks function as a centralized regulatory circuit that balances growth and coordinates subterranean resource acquisition. Deciphering the spatial and temporal dynamics of these multi-hormonal nodes wherein ABA, auxin, and ethylene pathways intersect is essential for engineering resilient root ideotypes that maintain productivity under fluctuating soil water potentials.

### 4.2 Genetic regulation of RSA

**Figure 4:**
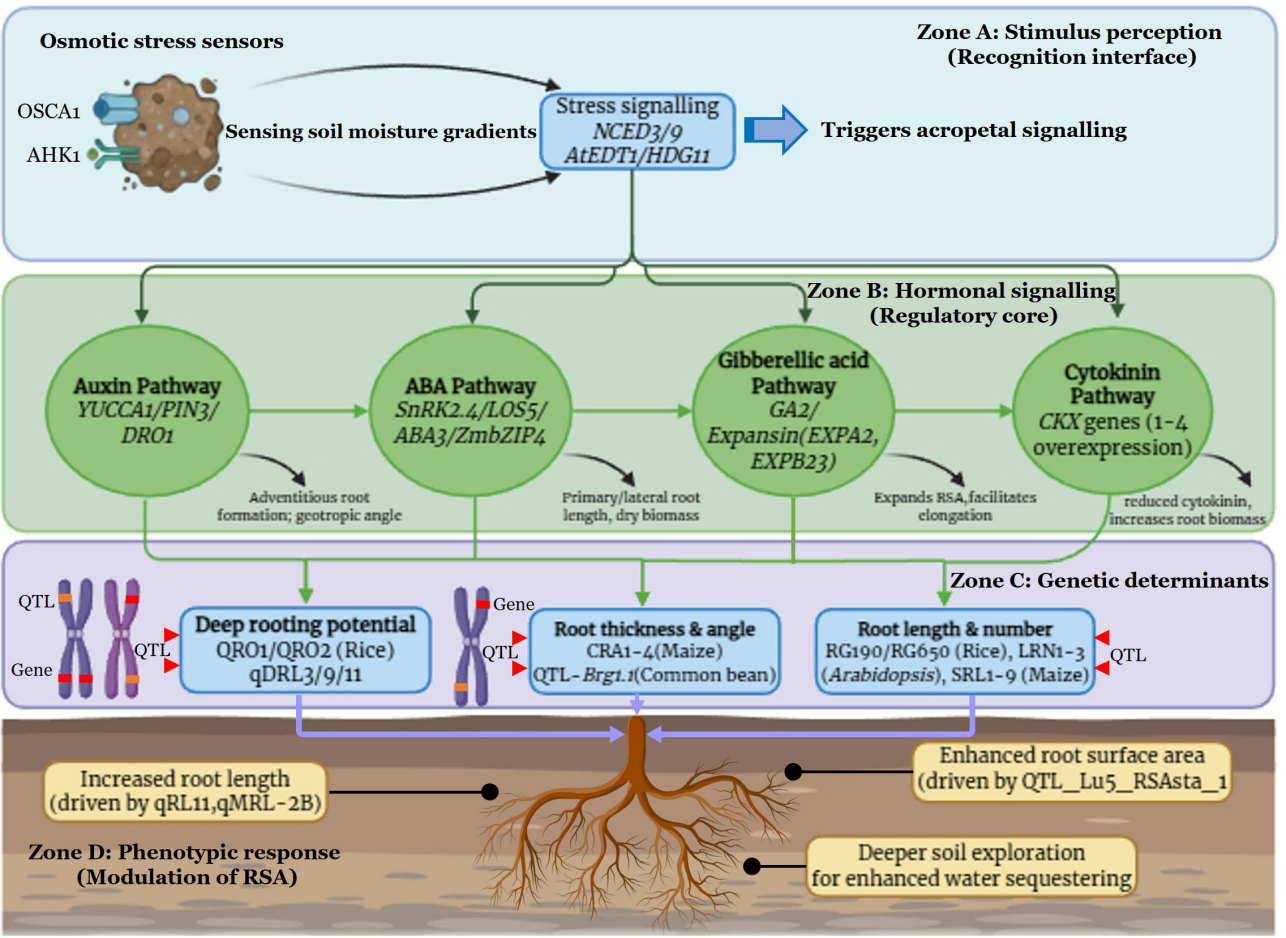
Hierarchical coordination of phytohormonal signaling and genetic determinants governing root adaptation to drought stress.

The genetic regulation of root architecture is a critical factor in enabling plants to adapt and mitigate the negative effects of abiotic stresses. The present section summarizes the key genetic mechanisms underlying the shaping of RSA in normal and drought environments. Root cells can regulate a wide range of molecular responses in enduring unfavourable environmental cues. Several investigations have been conducted to elucidate the role of RSA, utilizing the model plant *Arabidopsis thaliana* along with cereals like rice, wheat, and maize. We have presented a regulatory network of key genes and QTLs regulating RSA under drought stress (**Figure 4**). This model hierarchically organizes the identified loci according to their functional roles in stress perception, phytohormone-mediated signalling, and genomic structural control, thereby illustrating the mechanism of adaptive root plasticity under drought. The details of stress-responsive genes affecting RSA through phytohormone-mediated signaling pathways for drought stress response in different crop plants are presented in **Table 4**. Numerous genes primarily belonging to *NAC*, *AUX/IAA*, *WRKY*, *AP2/ERF*, and *MYB* families were found to be differentially expressed under drought stress in the roots of rice (Abdirad et al., 2022). The fundamental regulatory mechanism governing RSA pivotally involves auxin signaling. Auxin transcriptionally regulates the differentiation of distal stem cells at all developmental stages and growth conditions involving the transcription factors PLETHORA (PLTs) and the homeodomain transcription factor WUSCHEL RELATED HOMEOBOX 5 (WOX5) (Drisch and Stahl, 2015). The intrinsic cellular concentration of auxin is governed by tryptophan-independent and dependent pathways (Mano and Nemoto, 2012), leading to the synthesis of indole-3-acetic acid (IAA), the primary natural auxin. In response to a low auxin gradient, AUX/IAA proteins bind to Auxin Response Factor (ARF) modules, leading to negative regulation of auxin response genes (Abel et al., 1995). While under high auxin concentration, AUX/IAA proteins undergo degradation by SCFTIR E3 ubiquitin ligase complex. SUMOylated ARF7 transcription factor enhances its interaction with its repressor IAA3 (indole-3-acetic acid, which in turn induces expression of *LBD16*, causing inhibition of lateral root initiation under the moisture-deprived root growth zones (Orosa-Puente et al., 2018). The polar auxin transport under gravistimulation is governed by the PIN-FORMED (PIN) transporter protein family. The movement of auxin is facilitated by the auxin influx carrier AUX1 and the auxin efflux carrier PIN2, mobilizing auxin to the lateral root cap and the elongation zone. Under drought-simulated conditions, ABA regulates the expression of PIN1 and PIN2; thus, increased auxin gradient inhibits root elongation in *Arabidopsis*, resulting in the gravitropic curvature of the root cap. However, a mutation in the negative gravitropic response (NGR), a plasma membrane protein, leads to the reversal of auxin flow and polarity of PIN proteins in *ngr* mutant lines (Ge and Chen, 2019). Further, overexpression of *OsYUCCA1*, the gene responsible for auxin biosynthesis, resulted in an increased number of lateral roots, root hairs, and crown roots in rice (Yamamoto et al., 2007). The availability of auxin-transport mutants in crop plants (Forestan and Varotto, 2012) provides an opportunity to test the physiological significance of these processes in mediating root growth in drying soil. Further, overexpression of *OsAUX1* in rice (Zhao et al., 2016) exhibited precocious lateral root development under drought stress. A WUSCHEL-related homeobox gene: *PagWOX11/12a*, promoted root elongation in response to water stress (Wang et al., 2020). The DEEPER ROOTING 1 (*DRO1*) gene and its orthologs govern root system architecture by orienting root growth toward a steeper angle, a process fundamentally driven by the modulation of gravitropic set-point angles (GSA). In rice, *DRO1* is expressed in the root meristem and negatively regulated by auxin; its presence alters the distribution of auxin in the root tip, increasing the downward curvature (gravitropism) of the seminal roots (Uga et al., 2013; Kitomi et al., 2020). This ‘deeper rooting’ phenotype is achieved because *DRO1* shifts the GSA, causing roots to perceive and respond to gravity more acutely, thereby prioritizing vertical elongation over lateral expansion. Similarly, qSOR1, a DRO1 ortholog, maintains auxin gradients in the root cap to regulate soil-surface rooting, demonstrating that the *IGT* gene family dictates the vertical versus horizontal distribution of the root mass (Waite et al., 2020). Beyond gravitropism, deeper rooting is further enhanced by hydrotropism, where the ABA-signaling cascade and *MIZ1* trigger an asymmetric distribution of Ca2^+^ and cytokinin. This hormonal asymmetry induces differential cell elongation in the root cortex, actively directing the root tip toward deeper, moisture-rich soil layers (Tanaka-Takada et al., 2019; Chang et al., 2019). A high rate of allelic variations in the *OsMIZ1* gene has been identified specifically in *indica* rice. Further, higher gene expression levels positively correlated with the drought-tolerant genotypes in rice (Kaur et al., 2020). Like *miz1*, the *miz2* mutants exhibit similar phenology and root gravitropism (Miyazawa et al., 2009). Moreover, *Arabidopsis* mutants with cytokinin response defects showed increased root branching, leading to a higher survival rate under drought stress (Mason et al., 2005; Riefler et al., 2006; To and Kieber, 2008). A key regulator of the ethylene signaling pathway, Ethylene response factors (ERFs), particularly ERF2, are known to regulate embryonic root growth through the modulation of cellular ethylene and ABA. Underlying Jasmonic acid signalling in response to drought stress, sensed by CORONATINE INSENSITIVE1 (COI1)– JASMONATE ZIM-DOMAIN (JAZ) co-receptor, along with basic helix–loop–helix (bHLH) transcription factors MYC2, MYC3, and MYC4, inhibits primary root growth. Contrarily, bHLH transcription factors (bHLH3 and bHLH17) bind to JAZ proteins, which subsequently block the inhibition of root growth that is usually mediated by jasmonate (Han et al., 2023). Further, JAZ proteins antagonize the ROOT HAIR DEFECTIVE (RHD6) and RHD6 LIKE1 (RSL1) transcription factors, regulating the jasmonate-induced extension of root hair growth (Han et al., 2020). Moreover, upregulation of the *JIOsPR10* (Jasmonic acid inducible pathogenesis-related class 10) gene was found to be involved in root growth and development in *Oryza sativa* by activating stress-related proteins (Wu et al., 2016). In addition, EXPB7 (He et al., 2015) and WOX11 (Cheng et al., 2016) regulate root hair formation, thereby contributing significantly to increased drought resistance. The overexpression of Triptychon (TRY) transcription factors from the halophyte *Limonium bicolor* in *Arabidopsis* suggested their function in the pathway for salt-triggered root hair development (Leng et al., 2020). In Maize, overexpression of *ZmTIP1*, an S-acyltransferase encoding gene, regulates root hair length and enhances drought tolerance (Zhang et al., 2020a). However, *GLABRA2* TF negatively regulates root hair growth under osmotic stress (Wang et al., 2020). Overexpression of the *OsNAC045* transcription factor (crucial for lateral root development) exhibited enhanced survival capacity following exposure to both drought and salt conditions (Zheng et al., 2009), while overexpression of *OsNAC10* under transcriptional control of a root-specific promoter exhibited enhanced yield and survival compared with non-transformed plants in response to drought stress (Jeong et al., 2010). Rice transgenics were found to possess improved root growth and drought tolerance through the integration of *BRX* (*BREVIS RADIX-like*) (Liu et al., 2010a), *OsVP1*(H+ pyrophosphatase in tonoplasts) and *OsNHX1* genes (Na+/H+ exchangers) (Liu et al., 2010b). Deciphering the molecular interplay underlying RSA holds significant potential for securing crop productivity despite varied rhizosphere environments and stress exposures.

**Table 4:** Stress-responsive genes altering root traits through phytohormone-mediated signaling pathways conferring drought tolerance in different crop plants.

Hormonal pathway	Crops	Genes	Expression	Functions	References
Auxin	*Oryza sativa*	*YUCCA1*	Overexpression	Increased number of adventitious roots	Yamamoto et al., 2007
		*GH3.13*	Overexpression		Zhang et al., 2009
		*PIN3t*	Overexpression	Enable polar auxin transport, altering the performance of root growth	Zhang et al., 2012
		*GH3.2*	Overexpression	Increased number of adventitious roots	Du et al., 2012
		*DRO1*		Increased root length and regulation of root growth angle	Uga et al., 2011, 2013
	*A. thaliana*	*YUCCA6*	Overexpression	Enhanced auxin levels result in increased root hair formation	Ke et al., 2015
Cytokinin	*A. thaliana*	*CKX* *CKX2* *CKX3* *CKX4*	Overexpression	Reduced CK level and increased root biomass	Werner et al., 2010
		*CKX1*	Overexpression	Reformed root architecture and drought resistance	Pospíšilová et al., 2000
Gibberellic acid	*O. sativa*	*GA2ox*	Overexpression	Expanded root system and increased yield	Lo et al., 2017
	*Triticum aestivum*	*EXPA2*	Overexpression	Increased primary as well as lateral roots under drought stress	Yang et al., 2020
		*EXPB23*	Overexpression	Increased water retention capacity, hence improving root elongation	Han et al., 2012
		*LRD*	RNAi-mediated gene silencing	Enhanced lateral root density and number	Placido et al., 2020
	*Zea mays*	*JAZ14*	Overexpression	Confers drought tolerance by inducing crosstalk among the GA, ABA, and JA signaling pathways, and increases the number of lateral roots and root length	Zhou et al., 2015
Abscisic acid	*A. thaliana*	*AHK1*/histidine kinase 1	Overexpression	Positive regulator of osmo-sensing and root development	Tran et al., 2007
		*NCED3/9* (cis epoxycarotenoid dioxygenase)	Overexpression	Involves root-shoot signaling	Takahashi et al., 2018
		*AtEDT1/HDG11*	Overexpression	Increase proline content and a well-developed root system	Yu et al., 2016
		*OSCA1*	Overexpression	Membrane protein-mediated osmotic stress responses, and hence, root growth	Yuan et al., 2014
		*SWEET17*	Overexpression	Increased lateral root growth and density under drought	Valifard et al., 2021
	*O. sativa*	*AtEDT1/HDG11*	Overexpression	Deep and extensive root system	Yu et al., 2013
		*SNAC1*	Overexpression	Increased the fresh and dry weight of roots	Saad et al., 2013
		*OsABA8ox2*	CRISPR-Cas9knockout	Increased root dry biomass	Zhang et al., 2020b
		*RRS1*	Overexpression	Primary and lateral root length; root density	Gao et al., 2023
	*T. aestivum*	*SnRK2.4*	Overexpression	Increased primary roots and hence yield	Mao et al., 2010
	*Glycine max*	*LOS5/ABA3*	Overexpression	Increased root dry biomass	Li et al., 2013
	*Z. mays*	*ZmbZIP4*	Overexpression	Increased lateral root number and primary root length	Ma et al., 2018
		*ZmPTF1*	Overexpression	Higher lateral roots number; Increased seminal roots and lateral root length	Li et al., 2019
Ethylene	*O. sativa*	*JERF1*	Overexpression	Maintain the root growth	Zhang et al., 2010
	*O. sativa*	*OsDOF15*	Overexpression	Primary root elongation	Qin et al., 2019
Jasmonic acid	*O. sativa*	*JIOsPR10*	Overexpression	Involved in root growth and development by activating stress-related proteins	Wu et al., 2016
	*T. aestivum*	*EXPB23*	Overexpression	Increased water retention capacity and decreased osmotic potential	Han et al., 2012
	*A. thaliana*	*ZmJAZ14*	Overexpression	Increased primary and lateral root growth	Zhou et al., 2015
		*RHD6*	Overexpression	Root hair growth development	Han et al., 2020
		*RSL1*			
Salicylic acid	*N. tabacum*	*TaEXPA2*	Overexpression	Higher germination rate, longer primary as well as lateral roots	Chen et al., 2016

LEA, Late embryogenesis abundant; *JIOsPR10*, Jasmonic acid inducible pathogenesis-related class 10; P5CS, pyrroline-5-carboxylate synthase; PDH, Proline dehydrogenase; BR, Brassinosteroids.

### 4.3 QTL association and marker-assisted selection

Numerous investigations across diverse plant species have identified several Quantitative Trait Loci (QTLs) associated with the genetic regulation of RSA under moisture-deficient environments (**Supplementary Table 2**). These research findings have played a crucial role in advancing understanding for designing environment-specific root systems (Yadav et al., 2020; 2024a, 2024b). For instance, a major-effect QTL for root depth on rice chromosome 9 remains a primary target for improving yield under water-limiting conditions (Steele et al., 2006, 2007). Central to this regulation is the DEEPER ROOTING 1 (DRO1) locus; functional validation of Dro1-near-isogenic lines (NILs) confirms that this locus optimizes root growth angle to facilitate deep-soil water acquisition, thereby maintaining grain filling and reducing seed sterility under drought (Uga et al., 2013; Kitomi et al., 2020). These improvements are specifically attributed to vertical root distribution rather than shifts in other morphological traits (Arai-Sanoh et al., 2014). Furthermore, overlapping QTLs for root development, water-use efficiency, and nutrient absorption—including those regulating ABA-induced lateral root inhibition in Arabidopsis (Fitz et al., 2006; Xiong et al., 2006) underscore the pleiotropic nature of RSA in determining crop productivity (Johnson et al., 2000; Tuberosa et al., 2002). Despite these discoveries, the low heritability and high QTL × Environment (Q×E) interactions of root traits have historically limited their successful introgression. To resolve this, current research has shifted toward Meta-QTL (MQTL) analysis, which identifies stable, high-confidence genomic regions across varying genetic backgrounds and stress intensities (Selamat and Nadarajah, 2021; Kumar et al., 2020). Moreover, the integration of haplotype-based breeding and Genomic Selection (GS), supported by high-throughput phenotyping, now enables the simultaneous selection of minor-effect loci, thereby moving beyond the limitations of single-marker MAS (Sinha et al., 2023). Consequently, fine-mapping these stable MQTLs and identifying candidate genes remains pivotal for precision breeding of climate-smart varieties.

## 5 Conclusion and future perspectives

Research into root system architecture (RSAs) faces critical barriers that limit the translation of genetic insights into climate-resilient crops. Understanding and harnessing deep soil-based resources could be instrumental in attaining sustainable crop productivity in varied extreme growth environments. Significant progress has been made in understanding the functioning of different root traits in water acquisition by plants, and several root-specific QTLs have been identified, but defining an ideal RSA under a target environment still remains as an important challenge. The main problem remains that roots are by their nature difficult to phenotype in a non-destructive way under realistic field conditions, which creates a gap between controlled laboratory findings and the application in breeding. Moreover, the lack of standardized protocols and high phenotypic variability between environments complicates cross-study comparison. However, emerging technologies based on robust and high-throughput phenotyping platforms enabling accurate and real-time measurement offer promising solutions. Further, genomic approaches focused on highly heritable traits, such as root angle, depth, and xylem anatomy, in combination with marker-assisted selection, can speed up the propagation of drought-adaptive phenotypes. Future progress requires coordinated multi-environmental studies, open phenotypic data sets, and the integration of genetic selection. The combination of advanced phenotyping, systems biology, and precision agronomy will transform RSA knowledge into stress-tolerant varieties, ultimately securing global food systems in a changing climate.

Robust and high-throughput phenotyping platforms enabling accurate and real-time measurement will help fully realize this potential and thus adaptation to water- and resource-limited environments.
