# Supplementary material for: Root system architecture and drought adaptation: emerging tools and genetic insights
Source: Front Plant Sci. 2026 Jan 30;17:1753086. doi: 10.3389/fpls.2026.1753086 (PMC12901346; doi:10.3389/fpls.2026.1753086)
Supplement: Supplementary file 1 [file Table1.docx]

**Supplementary materials**

**Supplementary Table 1. Root traits showing functional significance for contribution to enhanced productivity under water-limited environments**

| **Root traits** | **Functional characteristics** | **Reference** |
| --- | --- | --- |
| Total root length/surface area | Total root system size in contact with soil | Sharma et al*.,* 2011; Wasson et al*.,* 2012; Manju et al., 2019; Kaur et al., 2020 |
| Root length/weight density | The quantity of root length or weight per unit volume of soil, directly related to the rate of uptake of water/nutrients | Wasson et al*.,* 2012; Sharma et al*.,* 2011; Kashiwagi et al*.,* 2006; Lynch, 2013; Rathod et al., 2022 |
| Root number, volume | Physical strength, ability to permeate a large volume of soil, potential for root system architecture | Sharma et al.*,* 2011; Giuliani et al*.,* 2005; Courtois et al*.,* 2009 |
| Root diameter | Penetration ability, branching, and hydraulic conductivity | Wasson et al*.,* 2012; Hernández et al*.,* 2010; Comas et al*.,* 2013 |
| Deep root growth and seminal root number | Ability to absorb soil moisture and nutrients from deeper layers | Hamada et al*.,* 2012; Kashiwagi et al*.,* 2006; Kato et al*.,* 2013; MacMillan et al*.,* 2006 |
| Seminal root angle and number | Degree of branching, the potential for better root architecture | Christopher et al*.,* 2013;  Manschadi et al*.,* 2006; Uga et al., 2013 |
| Root branching | Potential for soil exploration | Giuliani et al*.,* 2005 |
| Root xylem vessels' size and abundance | Degree of water extraction per unit of root length, hydraulic conductivity | Wasson et al.*,* 2012; Hernández et al*.,* 2010; Comas et al*.,* 2013  Giuliani et al*.,* 2005 |
| Root hair | Nutrient and water absorption, root anchoring | Bates and Lynch, 2001; Wasson et al*.,* 2012; Han et al., 2016, 2020; Maqbool et al., 2022 |
| Root cap | Perception and response to water potential gradient, sensing of hydro-stimulation | Miyazawa et al*.* 2009;  Hamada et al*.,* 2012 |

**Supplementary Table 2: Major QTLs for better root system architecture under drought stress in different crops**

| **Crops** | **QTL** | **Root trait** | **References** |
| --- | --- | --- | --- |
| Rice | RG190  RG650  G1085 | Root length | Prince et al., 2015a |
|  | RG83  RZ390  RZ70  G338 | Percent root mass | MacMillan et al., 2006 |
|  | *qDRM1.11* | Root biomass | Bhattarai and Subudhi, 2018 |
|  | C701 | Maximum root length | MacMillan et al., 2006 |
|  | RG690B-RZ730  RG667-RZ404  RG181-RG95 |  | Courtois et al., 2003 |
|  | RZ19-RG690  RG171-RG157  CDO418-RZ978  RZ12-RM201 |  | Shen et al., 2001 |
|  | RZ19-RG690  CDO418-RZ978 | Total root weight | Shen et al., 2001 |
|  | RG690-RZ730  CDO418-RZ978  RZ206-RZ422 | Deep root weight |  |
|  | RM229 | Maximum root thickness | MacMillan et al., 2006 |
|  | RZ801-RZ14  RZ69-RG449  RZ2337C-RM234 | Maximum root thickness | Courtois et al., 2003 |
|  | RZ649E-RZ801  RZ536B-RZ284  CDO365-RM21 | Total Root Weight | Courtois et al., 2003 |
|  | S1746/RG109B  L16/CDO718  RG650/CDO497  RG570/RG667 | Seminal Root Length | Zheng et al., 2003 |
|  | AAG-CAG1/RG462  RG409/T17  RG313/E1328  AAC-CTT10/RZ140  RZ588/RG472 | Lateral Root Length |  |
|  | RG191/AAC-CAG5  RM252/AGG-CAG7  AAC-CAG7/AAC-CTT10 | Lateral Root Number |  |
|  | RG109B/RM315  CDO920/BCD134  RM282/RZ574  RZ399/RZ448  RM328/RG570 | Adventitious Root Number |  |
|  | RG246-K5  RG104-RG348 | Total Root Number | Zheng et al., 2000 |
|  | *qRL11, qRFW9, qRFW11* | Root length, root density, root biomass, Deep root | Han et al., 2018 |
|  | *qRT9.1*  *qRT5.1* | Root length and Root number | Catolos et al., 2017 |
|  | *QRO1*  *QRO2* | Deep root phenotype | Kitomi et al., 2018 |
|  | *qDTY3.2* | Deeper root growth | Grondin et al., 2018 |
|  | *qDRL3*  *qDRL9*  *qDRL11* | Deep rooting length | Sabar et al., 2019 |
|  | *qDRV3* | Deep root volume |  |
|  | *qDRD3.1*  *qDRD3.2* | Deep root diameter |  |
|  | *qDRSA3.1*  *qDRSA3.2* | Deep root surface area |  |
|  | *QTL2,*  *QTL7*  *QTL9*  *QTL11* | Enhanced root length and thickness | Steele et al., 2006 |
| *Arabidopsis* | *qEDG1*  *qEDG2* | Root system morphology & Lateral root formation | Fitz Gerald et al., 2006 |
|  | *LRN1*  *LRN2*  *LRN3* | Lateral root number | Loudet et al., 2005 |
|  | *LRD1*  *LRD2*  *LRD3*  *LRD4* | Lateral root density |  |
|  | *LRL1*  *LRL2*  *LRL3* | Lateral root system |  |
| Maize | *CRA1*  *CRA2*  *CRA3*  *CRA4* | Crown root angle | Li et al., 2018 |
|  | *CRD1*  *CRD2* | Crown root diameter |  |
|  | *CRL1*  *CRL2*  *CRL3*  *CRL4* | Crown root length |  |
|  | *CRN1*  *CRN2* | Crown root number |  |
|  | *SRL1*  *SRL2*  *SRL3*  *SRL4*  *SRL5*  *SRL6*  *SRL7*  *SRL8*  *SRL9* | Seminal root length |  |
|  | *SRN1*  *SRN2*  *SRN3* | Seminal root number |  |
|  | *umc53a*  *bnl8.01* | Lateral root elongation | Ruta et al., 2010 |
|  | *umc53a*  *csu134d (thf)*  *umc107b (croc)* | Elongation rate of axile roots |  |
|  | *qSRA-6A* | Root length and Root biomass | Alahmad et al., 2019 |
|  | *qSRN.cgb-2B* | Seminal root number | Liu et al., 2013 |
|  | *qMRL-2B*  *qMRL-7B1* | Maximum root length | Ren et al., 2012 |
|  | *qPRL-2B1*  *qPRL-2B2*  *qPRL-7B* | Primary root length |  |
|  | *qLRL-1A*  *qLRL-2B*  *qLRL-4B*  *qLRL-6A*  *qLRL-6B1*  *qLRL-6B2* | Lateral root length |  |
|  | *qTRL-2B*  *qTRL-4B*  *qTRL-6A*  *qTRL-6B*  *qTRL-6D* | Total root length |  |
|  | *qRN-3A*  *qRN-3D*  *qRN-4B*  *qRN-6A*  *qRN-6B*  *qRN-6D* | Root tip number |  |
|  | *QRA.qgw-2A*  *QRA.qgw-3D*  *qRA.qgw-5D*  *QRA.qgw-6A*  *qRA.qgw-6B.1*  *QRA.qgw-6B.2* | Seminal Root Angle | Christopher et al., 2013 |
|  | *qRN.qgw-1B*  *qRN.qgw-3A*  *qRN1.qgw-3B*  *QRN.qgw-4A.1*  *qRN.qgw-4A.2*  *QRN.qgw-6A* | Seminal Root Number |  |
| Soybean | *Q_root_Gm01*  *Q_root_Gm03*  *Q_root_Gm04*  *Q_root_Gm08*  *Q_root_Gm20* | Fibrous rooting | Abdel-Haleem et al., 2011 |
|  | *TRL_Gm06*  *RDL3_Gm07*  *TRL_Gm08* | Root length | Prince et al., 2015b |
|  | *LRN_Gm08* | Lateral root number | Manavalan et al., 2015 |
| Sorghum | *qRA1_5*  *qRA2_5*  *qRA1_8*  *qRA1_10* | Nodal root angle | Mace et al., 2012 |
|  | *qRDW1_2*  *qRDW1_5*  *qRDW1_8* | Associated with root dry weight |  |
| Barley | *QRl.S42.2H*  *QRl.S42.3H*  *QRl.S42.5H* | Enhanced root length | Arifuzzaman et al., 2014 |
|  | *QRdw.S42.1H.a*  *QRdw.S42.1H.b*  *QRdw.S42.2H*  *QRdw.S42.3H*  *QRdw.S42.4H*  *QRdw.S42.5H*  *QRdw.S42.7H* | Associated with root dry weight |  |
| Chickpea | *QR3rld01* | Root length density | Jaganathan et al., 2015 |
|  | *CaLG06* | Root surface area |  |
|  | *QR3rtr01* | Root dry weight ratio |  |
| Pea | *rl1*  *rl2*  *rl3* | Root length | Fondevilla et al., 2010 |
| Common bean | *QTL-Brg1.1*  *QTL-Brg5.1*  *QTL-Brg5.2* | Basal root angle | Liao et al., 2004 |
| Rapeseed | *qRL-9-1*  *qRL-11-1*  *qRL-11-2*  *qRL-11-3*  *qRL-19-1* | Root length | Gad et al., 2021 |
| Sesame | *qRRL1*  *qRRL3-1*  *qRRL3-2*  *qRRL7*  *qRRL12* | Relative root length | Liang et al., 2021 |
| Flax | *QTL_Lu4_TRLsta_1*  *QTL_Lu6_TRLsta_2* | Total root length stability | Soto-Cerda et al., 2020 |
|  | *QTL_Lu5_TRVsta_1*  *QTL_Lu6_TRVsta_2* | Total root volume stability |  |
|  | *QTL_Lu5_RSAsta_1*  *QTL_Lu6_RSAsta_2* | Root surface area stability |  |
|  | *QTL_Lu11_14898826*  *QTL_Lu12_1386203*  *QTL_Lu12_4334157*  *QTL_Lu13_19862281*  *QTL_Lu14_15927781*  *QTL_Lu14_3980668*  *QTL_Lu2_21668160*  *QTL_Lu6_17695007*  *QTL_Lu9_19469655*  *QTL_Lu9_6368499* | Root development | Soto-Cerda et al., 2023 |
