# Supplementary material for: Root system architecture and drought adaptation: emerging tools and genetic insights
Source: Front Plant Sci. 2026 Jan 30;17:1753086. doi: 10.3389/fpls.2026.1753086 (PMC12901346; doi:10.3389/fpls.2026.1753086)
Supplement: Supplementary file 2 [file Supplementaryfile1.docx]

**References**

Abdel-Haleem, H., Lee, G. J., and Boerma, R. H. (2011). Identification of QTL for increased fibrous roots in soybean. *Theor. Appl. Genet.* 122, 935–946. [doi: 10.1007/s00122-010-1500-9](https://doi.org/10.1007/s00122-010-1500-9)

Alahmad, S., El Hassouni, K., Bassi, F. M., Dinglasan, E., Youssef, C., Quarry, G., et al. (2019). A major root architecture QTL responding to water limitation in durum wheat. *Front. Plant. Sci.* 10, 436. doi: [10.3389/fpls.2019.00436](https://doi.org/10.3389/fpls.2019.00436)

Arifuzzaman, M., Sayed, M. A., Muzammil, S., Pillen, K., Schumann, H., Naz, A. A., et al. (2014). Detection and validation of novel QTL for shoot and root traits in barley (*Hordeum vulgare* L.).  *Mol. Breed.* 34, 1373–1387. [doi: 10.1007/s11032-014-0122-3](https://doi.org/10.1007/s11032-014-0122-3)

Bates, T. R., and Lynch, J. P. (2001). Root hairs confer a competitive advantage under low phosphorus availability. *Plant Soil* 236, 243–250. [doi: 10.1023/A:1012791706800](https://doi.org/10.1023/A:1012791706800)

Bhattarai, U., and Subudhi, P. K. (2018). Identification of drought responsive QTLs during vegetative growth stage of rice using a saturated GBS-based SNP linkage map. *Euphytica* 214, 38. [doi: 10.1007/s10681-018-2117-3](https://doi.org/10.1007/s10681-018-2117-3)

Catolos, M., Sandhu, N., Dixit, S., Shamsudin, N. A. A., Naredo, M. E. B., McNally, K. L., et al. (2017). Genetic loci governing grain yield and root development under variable rice cultivation conditions. *Front. Plant Sci.* 8, 1763. [doi: 10.3389/fpls.2017.01763](https://doi.org/10.3389/fpls.2017.01763)

Christopher, J., Christopher, M., Jennings, R., Jones, S., Fletcher, S., Borrell, A., et al. (2013). QTL for root angle and number in a population developed from bread wheats (*Triticum aestivum*) with contrasting adaptation to water-limited environments. *Theor. Appl. Genet.* 126, 1563–1574. [doi: 10.1007/s00122-013-2074-0](https://doi.org/10.1007/s00122-013-2074-0)

Comas, L. H., Becker, S. R., Cruz, V. M. V., Byrne, P. F., and Dierig, D. A. (2013). Root traits contributing to plant productivity under drought. *Front. Plant Sci.* 4, 1–16. [doi: 10.3389/fpls.2013.00442](https://doi.org/10.3389/fpls.2013.00442)

Courtois, B., Ahmadi, N., Khowaja, F., Price, A. H., Rami, J. F., Frouin, J., et al. (2009). Rice root genetic architecture: meta-analysis from a drought QTL database. *Rice* 2, 115–128. [doi: 10.1007/s12284-009-9028-9](https://doi.org/10.1007/s12284-009-9028-9)

Courtois, B., Shen, L., Petalcorin, W., Carandang, S., Mauleon, R., and Li, Z. (2003). Locating QTLs controlling constitutive root traits in the rice population IAC *165 × Co39*. *Euphytica* 134, 335–345. [doi: 10.1023/B:EUPH.0000004987.88718.d6](https://doi.org/10.1023/B:EUPH.0000004987.88718.d6)

Fitz, G. J. N., Lehti-Shiu, M. D., Ingram, P. A., Deak, K. I., Biesiada, T., and Malamy, J. E. (2006). Identiﬁcation of quantitative trait loci that regulate *Arabidopsis* root system size and plasticity*. Genetics* 172, 485–498. [doi: 10.1534/genetics.105.047555](https://doi.org/10.1534/genetics.105.047555)

Fondevilla, S., Fernández-Aparicio, M., Satovic, Z., Emeran, A. A., Torres, A. M., Moreno, M. T., et al. (2010). Identification of quantitative trait loci for specific mechanisms of resistance to *Orobanche crenata* Forsk in pea (*Pisum sativum* L.). *Mol. Breed.* 25, 259–272. [doi: 10.1007/s11032-009-9330-7](https://doi.org/10.1007/s11032-009-9330-7)

Gad, M., Chao, H., Li, H., Zhao, W., Lu, G., and Li, M. (2021). QTL mapping for seed germination response to drought stress in *Brassica napus*. *Front. Plant Sci.* 11, 629970. [doi: 10.3389/fpls.2020.629970](https://doi.org/10.3389/fpls.2020.629970)

Giulani, S., Sanguineti, M. C., Tuberosa, R., Bellotti, M., Salvi, S., and Landi, P. (2005). Root-ABA_1_, a major constitutive QTL, affects maize root architecture and leaf ABA concentration at different water regimes. *J. Exp. Bot.* 56, 3061–3070. [doi: 10.1093/jxb/eri303](https://doi.org/10.1093/jxb/eri303)

Grondin, A., Dixit, S., Torres, R., Venkateshwarlu, C., Rogers, E., Mitchell-Olds, T., et al. (2018). Physiological mechanisms contributing to the QTL qDTY3.2 effects on improved performance of rice Moroberekan×Swarna BC_2_F_3:4_ lines under drought. *Rice* 11, 43. [doi: 10.1186/s12284-018-0234-1](https://doi.org/10.1186/s12284-018-0234-1)

Hamada, A., Nitta, M., Nasuda, S., Kato, K., Fujita, M., Matsunaka, H., et al. (2012). Novel QTLs for growth angle of seminal roots in wheat (*Triticum aestivum* L.). *Plant Soil* 354, 395–405. [doi: 10.1007/s11104-011-1075-5](https://doi.org/10.1007/s11104-011-1075-5)

Han, J., Shin, N., Jang, S., Yu, Y., Chin, J. H., and Yoo, S. C. (2018). Identification of quantitative trait loci for vigorous root development under water-deficiency conditions in rice. *Plant Breed. Biotech.* 6, 147–158. [doi: 10.9787/PBB.2018.6.2.147](http://dx.doi.org/10.9787/PBB.2018.6.2.147)

Han, X., Zhang, M., Yang, M., and Hu, Y. (2020). *Arabidopsis* JAZ proteins interact with and suppress RHD_6_ transcription factor to regulate jasmonate-stimulated root hair development. *Plant Cell* 32, 1049­–1062. [doi: 10.1105/tpc.19.00617](https://doi.org/10.1105/tpc.19.00617)

Han, Y., Xin, M., Huang, K., Xu, Y., Liu, Z., Hu, Z., et al. (2016). Altered expression of TaRSL_4_ gene by genome interplay shapes root hair length in allopolyploid wheat. *New Phytol.* 209, 721–732. [doi: 10.1111/nph.13615](https://doi.org/10.1111/nph.13615)

Hernández, E. I., Vilagrosa, A., Pausas, J. G., and Bellot, J. (2010). Morphological traits and water use strategies in seedlings of Mediterranean coexisting species. *Plant Ecol.* 207, 233–244. [doi: 10.1007/s11258-009-9668-2](https://doi.org/10.1007/s11258-009-9668-2)

Jaganathan, D., Thudi, M., Kale, S., Azam, S., Roorkiwal, M., Gaur, P. M., et al. (2015). Genotyping-by-sequencing based intra-specific genetic map refines a ‘QTL-hotspot’ region for drought tolerance in chickpea. *Mol. Genet. Genom.* 290, 559–571. [doi: 10.1007/s00438-014-0932-3](https://doi.org/10.1007/s00438-014-0932-3)

Kashiwagi, J., Krishnamurthy, L., Crouch, J. H., and Serraj, R. (2006). Variability of root length density and its contributions to seed yield in chickpea (*Cicer arietinum* L.) under terminal drought stress. *Field Crops Res.* 95, 171–181. [doi: 10.1016/j.fcr.2005.02.012](https://doi.org/10.1016/j.fcr.2005.02.012)

Kato, M., Aoyama, T., and Maeshima, M. (2013). The Ca^2+^-binding protein PCaP_2_ located on the plasma membrane, is involved in root hair development as a possible signal transducer. *Plant J.* 74, 690–700. [doi: 10.1111/tpj.12155](https://doi.org/10.1111/tpj.12155)

Kaur, V., Yadav, S. K., Wankhede, D. P., Pulivendula, P., Kumar, A., and Chinnusamy, V. (2020). Cloning and characterization of a gene encoding *MIZ1*, a domain of unknown function protein and its role in salt and drought stress in rice. *Protoplasma* 257, 475–487. [doi: 10.1007/s00709-019-01452-5](https://doi.org/10.1007/s00709-019-01452-5)

Kitomi, Y., Nakao, E., Kawai, S., Kanno, N., Ando, T., Fukuoka, S., et al. (2018). Fine mapping of QUICK ROOTING 1 and 2, quantitative trait loci increasing root length in rice. G3 8, 727–735. doi: 10.1534/g3.117.300147

Li, P., Zhang, Y., Yin, S., Zhu, P., Pan, T., Xu, Y., et al. (2018). QTL-by-environment interaction in the response of maize root and shoot traits to different water regimes. *Front. Plant Sci.* 9, 229. [doi: 10.3389/fpls.2018.00229](https://doi.org/10.3389/fpls.2018.00229)

Liang. J., Sun, J., Ye, Y., Yan, X., Yan, T., Rao, Y., et al. (2021). QTL mapping of PEG-induced drought tolerance at the early seedling stage in sesame using whole genome re-sequencing. *PLoS One* 16, e0247681. [doi: 10.1371/journal.pone.0247681](https://doi.org/10.1371/journal.pone.0247681)

Liao, H., Yan, X., Rubio, G., Beebe, S. E., Blair, M. W., and Lynch, J. P. (2004). Genetic mapping of basal root gravitropism and phosphorus acquisition efficiency in common bean. *Funct. Plant Biol.* 31, 959–970. [doi: 10.1071/fp03255](https://doi.org/10.1071/fp03255)

Liu, X., Li, R., Chang, X., and Jing, R. (2013). Mapping QTLs for seedling root traits in a doubled haploid wheat population under different water regimes. *Euphytica*189, 51–66. [doi: 10.1007/s10681-012-0690-4](https://doi.org/10.1007/s10681-012-0690-4)

Loudet, O., Gaudon, V., Trubuil, A., and Daniel-Vedele, F. (2005). Quantitative trait loci controlling root growth and architecture in *Arabidopsis thaliana* confirmed by heterogeneous inbred family. *Theor. Appl. Genet.* 110, 742–753. [doi: 10.1007/s00122-004-1900-9](https://doi.org/10.1007/s00122-004-1900-9)

Lynch, J. P. (2013). Steep, cheap and deep: an ideotype to optimize water and N acquisition by maize root systems. *Ann. Bot.* 112, 347–357. [doi: 10.1093/aob/mcs293](https://doi.org/10.1093/aob/mcs293)

Mace, E. S., Singh, V., Van Oosterom, E. J., Hammer, G. L., Hunt, C. H., and Jordan, D. R. (2012). QTL for nodal root angle in sorghum (*Sorghum bicolor* L. Moench) co-locate with QTL for traits associated with drought adaptation. *Theor. Appl. Genet.* 124, 97–109. [doi: 10.1007/s00122-011-1690-9](https://doi.org/10.1007/s00122-011-1690-9)

MacMillan, K., Emrich, K., Piepho, H. P., Mullins, C. E., and Price, A. H. (2006). Assessing the importance of genotype × environment interaction for root traits in rice using a mapping population II: conventional QTL analysis. *Theor. Appl. Genet.* 113, 953–964. [doi: 10.1007/s00122-006-0357-4](https://doi.org/10.1007/s00122-006-0357-4)

Manavalan, L. P., Prince, S. J., Musket, T. A., Chaky, J., Deshmukh, R., Vuong, T. D., et al. (2015). Identification of novel QTL governing root architectural traits in an interspecific soybean population. *PLoS One* 10, e0120490. [doi: 10.1371/journal.pone.0120490](https://doi.org/10.1371/journal.pone.0120490)

Manju., Kaur, V., Sharma, K. D., and Kumar, A. (2019). Identification of promising sources for drought tolerance in cultivated and wild species germplasm of barley based on root architecture. J. Environ. Biol. 40 (3), 309-315. [doi: 10.22438/jeb/40/3/MRN-995](http://dx.doi.org/10.22438/jeb/40/3/MRN-995)

Manschadi, A. M., Christopher, J., deVoil, P., and Hammer, G. L. (2006). The role of root architectural traits in adaptation of wheat to water-limited environments. *Funct. Plant Biol.* 33, 823–837. [doi: 10.1071/fp06055](https://doi.org/10.1071/fp06055)

Maqbool, S., Saeed, F., Raza, A., Rasheed, A., and He, Z. (2022). Association of root hair length and density with yield-related traits and expression patterns of TaRSL_4_ underpinning root hair length in spring wheat. *Plants* 11, 2235. [doi: 10.3390/plants11172235](https://doi.org/10.3390/plants11172235)

Miyazawa, Y., Ito, Y., Moriwaki, T., Kobayashi, A., Fujii, N., and Takahashi, H. (2009). A molecular mechanism unique to hydrotropism in roots. *Plant Sci.* 177, 297–301. [doi: 10.1016/j.plantsci.2009.06.009](https://doi.org/10.1016/j.plantsci.2009.06.009)

Prince, S. J., Beena, R., Gomez, S. M., Senthivel, S., and Babu, R. C. (2015a). Mapping consistent rice (*Oryza sativa* L.) yield QTLs under drought stress in target rainfed environments. *Rice* 8, 53. doi: [10.1186/s12284-015-0053-6](https://doi.org/10.1186/s12284-015-0053-6)

Prince, S. J., Song, L., Qiu, D., dos Santos, J. V. M., Chai, C., Joshi, T., et al. (2015b). Genetic variants in root architecture-related genes in a *Glycine soja* accession, a potential resource to improve cultivated soybean. *BMC Genomics* 16, 132. [doi: 10.1186/s12864-015-1334-6](https://doi.org/10.1186/s12864-015-1334-6)

Rathod, G. R., Pandey, R., Chinnusamy, V., Paul, V., Jain, N., Singh, M. P., et al. (2022). Deeper root system architecture confers better stability to photosynthesis and yield compared to shallow system under terminal drought stress in wheat (Triticum aestivum L.). Plant Physiol. Rep. **27**, 250–259. [doi: 10.1007/s40502-022-00652-1](https://doi.org/10.1007/s40502-022-00652-1)

Ren, Y., He, X., Liu, D., Li, J., Zhao, X., Li, B., et al. (2012). Major quantitative trait loci for seminal root morphology of wheat seedlings. *Mol. Breed.* 30, 139–148. [doi: 10.1007/s11032-011-9605-7](https://doi.org/10.1007/s11032-011-9605-7)

Ruta, N., Liedgens, M., Fracheboud, Y., Stamp, P., and Hund, A. (2010). QTLs for the elongation of axile and lateral roots of maize in response to low water potential. *Theor. Appl. Genet.* 120, 621–631. [doi: 10.1007/s00122-009-1180-5](https://doi.org/10.1007/s00122-009-1180-5)

Sabar, M., Shabir, G., Shah, S. M., Aslam, K., Naveed, S. A., and Arif, M. (2019). Identification and mapping of QTLs associated with drought tolerance traits in rice by a cross between super basmati and IR55419-04. *Breed. Sci.* 69, 169–178. [doi: 10.1270/jsbbs.18068](https://doi.org/10.1270/jsbbs.18068)

Sharma, S., Xu, S., Ehdaie, B., Hoops, A., Close, T. J., Lukaszewski, A., et al. (2011). Dissection of QTL effects for root traits using a chromosome arm-speciﬁc mapping population in bread wheat. *Theor. Appl. Genet.* 122, 759–769. [doi: 10.1007/s00122-010-1484-5](https://doi.org/10.1007/s00122-010-1484-5)

Shen, L., Courtois, B., McNally, K. L., Robin, S., and Li, Z. (2001). Evaluation of near-isogenic lines of rice introgressed with QTLs for root depth through marker-aided selection. *Theor. Appl. Genet.* 103, 75–83. [doi: 10.1007/s001220100538](https://doi.org/10.1007/s001220100538)

Soto-Cerda, B. J, Cloutier, S., Gajardo, H. A., Aravena, G., Quian, R., and You, F. M. (2020). Drought response of flax accessions and identification of quantitative trait nucleotides (QTNs) governing agronomic and root traits by genome-wide association analysis. *Mol. Breed.* 40, 15. [doi: 10.1007/s11032-019-1096-y](https://doi.org/10.1007/s11032-019-1096-y)

Soto-Cerda, B. J., Larama, G., Cloutier, S., Fofana, B., Inostroza-Blancheteau, C., and Aravena, G. (2023). The genetic dissection of nitrogen use-related traits in flax (*Linum usitatissimum* L.) at the seedling stage through the integration of multi-locus GWAS, RNA-seq and genomic selection. *Int. J. Mol. Sci.* 24, 17624. [doi: 10.3390/ijms242417624](https://doi.org/10.3390/ijms242417624)

Steele, K. A., Price, A. H., Shashidhar, H. E., and Witcombe, J. R. (2006). Marker-assisted selection to introgress rice QTLs controlling root traits into an Indian upland rice variety. *Theor. Appl. Genet*. 112, 208–221. [doi: 10.1007/s00122-005-0110-4](https://doi.org/10.1007/s00122-005-0110-4)

Uga, Y.,  [Sugimoto](http://www.nature.com/ng/journal/v45/n9/full/ng.2725.html#auth-2), K.,  [Ogawa](http://www.nature.com/ng/journal/v45/n9/full/ng.2725.html#auth-3), S.,  [Rane](http://www.nature.com/ng/journal/v45/n9/full/ng.2725.html#auth-4), J.,  [Ishitani](http://www.nature.com/ng/journal/v45/n9/full/ng.2725.html#auth-5), M.,  [Hara](http://www.nature.com/ng/journal/v45/n9/full/ng.2725.html#auth-6), N., et al. **(**2013). Control of root system architecture by DEEPER ROOTING 1 increases rice yield under drought conditions. *Nat. Genet.* 45, 1097–1102. [doi: 10.1038/ng.2725](https://doi.org/10.1038/ng.2725)

Wasson, A. P., Richards, R. A., Chatrath, R., Misra, S. C., Prasad, S. V, Rebetzke, G. J., et al. (2012). Traits and selection strategies to improve root systems and water uptake in water-limited wheat crops. *J. Exp. Bot.* 63, 3485–3498. [doi: 10.1093/jxb/ers111](https://doi.org/10.1093/jxb/ers111)

Zheng, B. S., Yang, L., Zhang, W. P., Mao, C. Z., Wu, Y. R., Yi, K. K., et al. (2003). Mapping QTLs and candidate genes for rice root traits under different water-supply conditions and comparative analysis across three populations. *Theor. Appl. Genet.* 107, 1505–1515. [doi: 10.1007/s00122-003-1390-1](https://doi.org/10.1007/s00122-003-1390-1)

Zheng, H. G., Babu, R. C., Pathan, M. S., Ali, L., Huang, N., Courtois, B., et al. (2000). Quantitative trait loci for root-penetration ability and root thickness in rice: comparison of genetic backgrounds. *Genome* 43, 53–61. [doi: 10.1139/g99-065](https://doi.org/10.1139/g99-065)
